# A community health worker led approach to cardiovascular disease prevention in the UK—SPICES-Sussex (scaling-up packages of interventions for cardiovascular disease prevention in selected sites in Europe and Sub-saharan Africa): an implementation research project

**DOI:** 10.3389/frhs.2024.1152410

**Published:** 2024-05-07

**Authors:** Thomas Grice-Jackson, Imogen Rogers, Elizabeth Ford, Robert Dickinson, Kat Frere-Smith, Katie Goddard, Linda Silver, Catherine Topham, Papreen Nahar, Geofrey Musinguzi, Hilde Bastiaens, Harm Van Marwijk

**Affiliations:** ^1^ Department of Primary Care and Public Health, Brighton and Sussex Medical School, Brighton, United Kingdom; ^2^ Department of Global Health Infection, Brighton and Sussex Medical School, University of Sussex, Brighton, United Kingdom; ^3^ Department of Disease Control and Environmental Health, Makerere University, Kampala, Central Region, Uganda; ^4^ Faculty of Medicine and Health Sciences, University of Antwerp, Antwerp, Belgium

**Keywords:** community based participatory research, implementation research, RE-AIM (reach, effectiveness, adoption, implementation and maintenance), cardiovascular disease, community health workers (CHW)

## Abstract

**Background:**

This paper describes a UK-based study, SPICES-Sussex, which aimed to co-produce and implement a community-based cardiovascular disease (CVD) risk assessment and reduction intervention to support under-served populations at moderate risk of CVD. The objectives were to enhance stakeholder engagement; to implement the intervention in four research sites and to evaluate the use of Voluntary and Community and Social Enterprises (VCSE) and Community Health Worker (CHW) partnerships in health interventions.

**Methods:**

A type three hybrid implementation study design was used with mixed methods data. This paper represents the process evaluation of the implementation of the SPICES-Sussex Project. The evaluation was conducted using the RE-AIM framework.

**Results:**

Reach: 381 individuals took part in the risk profiling questionnaire and forty-one women, and five men participated in the coaching intervention. Effectiveness: quantitative results from intervention participants showed significant improvements in CVD behavioural risk factors across several measures. Qualitative data indicated high acceptability, with the holistic, personalised, and person-centred approach being valued by participants. Adoption: 50% of VCSEs approached took part in the SPICES programme, The CHWs felt empowered to deliver high-quality and mutually beneficial coaching within a strong project infrastructure that made use of VCSE partnerships. Implementation: Co-design meetings resulted in local adaptations being made to the intervention. 29 (63%) of participants completed the intervention. Practical issues concerned how to embed CHWs in a health service context, how to keep engaging participants, and tensions between research integrity and the needs and expectations of those in the voluntary sector. Maintenance: Several VCSEs expressed an interest in continuing the intervention after the end of the SPICES programme.

**Conclusion:**

Community-engagement approaches have the potential to have positively impact the health and wellbeing of certain groups. Furthermore, VCSEs and CHWs represent a significant untapped resource in the UK. However, more work needs to be done to understand how links between the sectors can be bridged to deliver evidence-based effective alternative preventative healthcare. Reaching vulnerable populations remains a challenge despite partnerships with VCSEs which are embedded in the community. By showing what went well and what did not, this project can guide future work in community engagement for health.

## 1 Introduction

Cardiovascular disease (CVD) is among the most prevalent, costly to treat, and deadly medical issues in the world (1). As part of the continual effort to combat CVD, greater emphasis is being placed on prevention. This often takes the form of behavioural or lifestyle change, focusing on the reduction of risk factors (e.g., hypertension, poor diet, obesity). Reducing these risk factors using evidence-based interventions not only works to lower rates of CVD, but also impacts rates of a variety of other medical issues, including susceptibility to severe COVID-19 infection (2), many common Noncommunicable Diseases (NCDs) including Type 2 diabetes and a wide range of cancers (3). Furthermore these preventative interventions are less expensive than reactionary care and can lower the treatment burden on strained medical systems (4).

Community-Based Participatory Research (CBPR) and Community Engagement (CE) have grown increasingly popular as potential methods to engender sustainable, long-term change in communities—particularly those communities under-served by existing medical systems and/or those at heightened risk of CVD (5). One's behaviour is influenced by their environment and the community they live in, meaning that tapping into a community's resources can be effective in changing lifestyle behaviour as well as having impacts on the wider community (6, 7). The use of community-based practices fits within the growing South-North collaboration that this project joins as part of an international collaboration known as “Scaling-up Packages of Interventions for Cardiovascular disease prevention in selected sites in Europe and Sub-Saharan Africa: An implementation research project” (SPICES). In the Low- and Middle-Income countries (LMIC) there is evidence for the successful implementation of evidence-based community-based interventions in increasing knowledge of, and changing behaviour related to, CVD (8) however their use in the Global North is less well tested or understood (9).

In the UK, the flagship intervention to address preventative health issues is the National Health Service's (NHS) Health Check initiative, which is free to individuals ages 40–76 and which assesses risk for long term health conditions including CVD (10). Following initial assessment by a health professional, patients are advised on a course of action which often includes some degree of preventative prescribing to address behavioural risk factors (11). Just under half of eligible individuals accepts a first health-checks appointment (44.2%)—it is associated with increased detection of CVD risk, but uptake is skewed by several demographic factors (principally, age, gender, and socio-demographics), and it has struggled to create change in underserved groups (12, 13). Marginalised coastal communities in Sussex face overall below-average healthy-life expectancy (14). This, alongside heightening inequality and the impact of COVID-19, has left some communities in Sussex significantly deprived in terms of access and engagement with health services (15). People in these communities experience transgenerational poverty, precarity, and lifestyle behaviours ingrained into the communities that lead many to be at higher risk for CVD. CBPR and CE models have the potential to lead to improved health and health behaviours among disadvantaged populations if designed properly and implemented through effective community consultation and participation (16).

CBPR and CE offer the chance to bring lessons from effective programmes in the Global South and apply them to programmes in the Global North. Community-based strategies to promote evidence-based preventative health interventions using Community Health Workers (CHWs) are often more established in the Global South where more tightly knit communities and established community health programmes fulfil a range of public health needs (17, 18). CHWs interventions are a form of “task-sharing” intervention in which responsibility and power is shared between professional health workers and communities which have been proposed to effectively manage non-communicable disease risk (19). Lay Community Health Workers are individuals who are trained to perform of health-related functions but lack a formal professional health education. They can provide links between local communities and health care institutions thereby building and on and developing the social capital that already exists in communities (20). Although there is plenty of evidence communicating the importance and usefulness of these methods (the “what”), there remains a lack of attention given to *how to do it*. This article joins the work and voices attempting to begin filling that lacuna.

Within the literature on CBPR and CE, a handful of common themes emerge. The first is a push for human-centred research design (21, 22). Yardley et al. (23) focused on this idea in their “person-based” approach to digital health interventions, where they recommended a “focus on understanding and accommodating the perspectives of the people who will use the intervention” (24). Hopkins and Rippon's (25) “asset-based” approach to CE interventions recommends recognising and adapting to the need, wants, and strengths already present in the community. Particularly the strengths, or “assets” already present in the community provide an opportunity for projects to use those assets. Such an implementation approach requires flexibility and adaptability, as well as deep involvement with the community. The second theme builds on the first, with the idea that not only should project design be person-centred, but those participants and other stakeholders in the community should be involved at every level of project planning through co-design. Yardley et al. (23) included this as a key element of their paper, writing that people from the target population should be involved in project development as well as at every stage of the intervention. Similarly, Berrera et al. (26) emphasise the need to adapt all projects to the cultural context of the community. This insight speaks to the third theme, continuous evaluation (27). As the needs of the community will be ever-shifting, so must the project adapt to those needs continually. Instead of designated periods of evaluation, a shift to continual processes of qualitative evaluation is called for to identify and adjust to the needs of the community. These processes require elevated levels of trust and participation from the community, which has its own challenges. Trust especially takes significant time and resources to develop and is an under-studied area of community engagement (28).

The SPICES-Sussex project was carried out from January 2019 and aimed to answer the following overarching research question: How can Community Health Workers (CHW) CVD prevention interventions, that have been used in the Global South, be developed, and implemented in a Global North setting and what barriers and enablers exist to their implementation? The project began with a situational analysis which included an exploration of the views and experiences of the local community with regards to CVD health and Community Health Workers and early stakeholder mapping of the research sites which was carried out between 2019 and 2020 (29, 30).

The primary aim of the current paper is to provide a comprehensive examination of the project's implementation including complementary mixed methods analyses according to the Reach Effectiveness-Adoption Implementation and Maintenance (RE-AIM) framework (31). The secondary aims of the project are to inform future CE projects what worked (and did not work) for our project and to tie insights from our project to broader discussions in the discipline. The project is based on a protocol published in 2020 prior to the onset of COVID and was conducted through the period of the COVID-19 pandemic (29). Subsequently, several aspects of the original protocol were adapted to make implementation feasible within the constraints of this period (see Supplementary Appendix 2).

## 2 Methods

### 2.1 Study design

The project uses a type 3 hybrid implementation design (29) meaning that the primary aim of the research was to determine utility of an implementation intervention/strategy whilst the secondary aim was to assess clinical outcomes associated with the implementation trial. This means that we focused on understanding what barriers and enablers existed for the project's implementation and the context within which it operated. Effectiveness of the intervention remained important, however we were primarily interested in how and why it did (or did not) work. The project was carried out at four geographic research sites within Sussex (see Section 2.3) and implementation was conducted on an iterative basis from research site to research site broadly following the Medical Research Council's (MRC) framework for the development and implementation of complex interventions (32). The research team developed and then began delivering the intervention at each site before moving onto the next. At each site the following stages were carried out: (1) Development: this included stakeholder mapping, formation of implementation partners, and codesign/local adaptation of the intervention [covered in the study's pre-implementation paper (30)]; (2) Implementation: this included the delivery of the CHW intervention at the research sites and collection of mixed method data pertaining to effectiveness and stakeholder experiences, and (3) Evaluation: this included the analysis of the mixed method data in line with the MRC guidance on analysis complex interventions.

### 2.2 Research site and voluntary and community sector enterprise partner selection

Four study sites were selected across East Sussex by identifying Middle Layer Super Output Area (MSOA) postcodes with high levels of deprivation according to the Indices of Multiple Deprivation (IMD) (33). Selection of the research sites was based on the pre-implementation community mapping phase of the project (30). Following on from CBPR practices, VCSEs and Volunteer Coordinators (VCs) were recruited to co-design and deliver the implementation strategy at each of the research sites.

VCSEs organisations were recruited as partners at each research site. The intervention was primarily run through these organisations and a paid staff member was recruited at each organisation. Their responsibilities included, CHW management, and participant recruitment. They also had a role in local adaptation activities. VCSE organisations were eligible to take part in the organisations if they were based in the research site, if they had interests and existing activities that aligned with the project's goals (CVD risk reduction and community development), and if they had existing experience of volunteer recruitment and management.

### 2.3 Community health worker recruitment and training

The aim was for each site to recruit a pool of five to eight CHWs. As part of this each site was asked for input into local CHW recruitment flyers, which were shared on VCSE websites and social media pages and shared on social media via existing CHWs at the VCSEs. CHWs were recruited through intermediary organisation recruitment via the VCSE partner organisation. The project was also advertised at a Virtual Volunteer Fair. Local contacts and existing volunteer pools at the VCSEs meant that the target number of CHWs was rapidly recruited at each site. CHWs were eligible to take part in the intervention if they were over 18 years of age, if they lived within the research site (determined by postcode), and if they had some kind of pre-existing relationship with the VCSE partner organisation (i.e., as a volunteer).

Potential CHWs who expressed an interest in the project were invited to attend an induction to the project, and then the local adaptation co-design meeting. Those who decided they would like to become a CHW then went on to receive five online, group training sessions (each of which lasted for 2 h, 10 h in total): an introductory session, a session covering project policies, heart health and the structure of the intervention, and three sessions on behaviour change techniques. These training sessions were developed and delivered by an external organisation (National Centre for Behaviour Change) specifically for the project after a consultation and planning process with the research team. Before the onset of the intervention at each site CHWs made various recommendations in the local adaptation meetings on the design of the training programme. These included providing information on listening techniques, engaging, and managing resistance, providing simple health information, using accessible language, using different starting points depending on the CHW's background knowledge and experience, training on conducting coaching virtually, and providing a training handbook. A Volunteer coordinator (VC) was recruited at each site. This VC was a trained and experience health coach (KFS) and provided training support and guidance through monthly group training support sessions in addition to the initial 10 h training block the received prior to the intervention onset. These monthly training and support sessions were organised into specific themes and agendas that were set with the CHW participants.

### 2.4 Local adaptation

Elements of the evidence-based intervention were tailored to the individuals and their community in the stakeholder-mapping phase using qualitative interviews, workshops, and focus groups with a range of stakeholders across the study site (30). Further rounds of local adaptation were carried out with VCs and CHWs at each of the research sites to tailor to individuals and their community context through iterative co-design workshops (34). CHWs and VCSE also agreed on a “volunteer charter” during the co-design session. This was a list of principles, behaviours, and practices upon which guided interactions between research staff, CHWs, VCSE and participants. The charter was designed to ensure that the practices of the project aligned with the principles of the CHW and partner organisation.

### 2.5 Participant recruitment and screening questionnaire

Participants (who received coaching) were eligible to take part in the eligibility screening if they lived in, or adjacent to, the study site's postcode and if they were aged eighteen or older. Participant recruitment was also based on intermediary organisation recruitment, community outreach, paid social media advertisement (through Meta™), gatekeeper and snowball sampling. Gatekeeper recruitment was conducted when interacting with a relevant statutory or non-statutory service provider (i.e., a fitness/weight loss group leader) and involved asking them to recommend the intervention to their members or to recommend participants who may be interested in taking part. Snowball sampling involved asking participants who participated in the study to sending email invitations to their social group. A social media recruitment strategy was undertaken to recruit people from the local area to the risk profiling survey to supplement the community-based recruitment through the VCSE partners. Social media was conducted on Facebook via paid advertisement in four waves of recruitment which took place over 1–2 weeks at each site. The advert targeted people who were 35+ and over and to people with 5 km of each research site. Messages were changed regularly from a list of recruitment messages drafted with CHWs during co-design sessions. Additionally, CHWs and VCSE participants were asked to send recruitment emails to any social or professional networks they thought would be interested in taking part. We did not record where participants were recruited.

Screening and risk profiling for the CVD coaching was carried out using the validated non-laboratory based INTERHEART questionnaire, presented online, for all participants that expressed an interest in the study (35). This questionnaire assessed modifiable and non-modifiable CVD risk factors and categorised participants as either “Low,” “Moderate.” Or “High” risk. See the protocol paper for further information on the INTERHEART risk profiling; for more information on the screening questionnaire, see the study protocol paper (29, 35). Questionnaire data were collected and managed using Research Electronic Data Capture (REDCap) electronic data capture tools hosted at the University of Antwerp (36). Participants were considered to be eligible for the intervention if they were aged eighteen or over, if they lived within the research site (determined by postcode), and if they were categorised as “Moderate” risk of CVD according to the INTERHEART questionnaire. High risk participants were not included as their needs were considered to be too high for a pilot study involving CHWs. Eligible participants were then emailed by the research team with an invitation to take part in the CVD coaching intervention. After recruitment for the intervention was closed for each site, an online questionnaire survey was sent to eligible participants to gather information the reasons for not accepting the invitation to the intervention. Open response questions were used which the research team later categorised into codes.

### 2.6 The CVD prevention coaching intervention

The coaching intervention was based on motivational interviewing techniques which are promoted by the European commission on cardiovascular disease prevention in clinical practice (37) and which include techniques such as Open questions, Affirmation, Reflective listening and Summary reflections (OARS) (38, 39). The use of these Behaviour Change Techniques (BCTs) used during the intervention were based on five target behaviours highlighted by the World Health Organisation including: reduce/cease smoking, increase moderate physical activity, reduce the fat, salt, and sugar content of the diet, increase fibre, oily fish (or alternatives), fruit, and vegetable content of the diet, reduce sedentary hours. The intervention involved six, one-hour long coaching sessions between participants and CHWs which were delivered every two weeks. Participants were also considered to have completed the intervention if they only completed three sessions and then notified the team of their withdrawal from the intervention.

The study team included two participant co-ordinators (PCs) who managed the participant journey through the intervention, sending welcome emails, questionnaires, and invitations to post-intervention interviews, and co-ordination between participants and CHWs to book coaching sessions. Reminders of appointments were also sent to CHWs and participants one week and two days before the session. Participants and CHWs were matched, based on gender preference and availability, and supported throughout the coaching intervention the PCs. CHWs were provided with guidance, resources, and signposting information throughout the intervention but were also given the flexibility to deliver the coaching in a way that suited them and their participant(s). Initially, counselling and goalsetting were based on their individual item INTERHEART assessment scores. Participants and CHWs were then encouraged to create an action plan with appropriate goal setting for the behaviours they wanted to change (e.g., diet, exercise habits). The goals were set in relation to when, where, and how they would undertake the behaviour, e.g., when the physical activity will be performed, where it will be performed, how often it will be performed (i.e., in a group or using specific equipment). CHWs helped participants to analyse any factors which might influence their ability to achieve the goals and to generate strategies which could help them overcome these barriers using problem solving. Full details of the participant journey through the intervention are given in Supplementary Appendix 2 in the Supplementary Material. All coaching was conducted virtually using Zoom™ to host and monitor coaching sessions and Microsoft OneDrive to store, recruit, and communicate written and visual resources with CHWs and participants. Monitoring in Zoom calls was called out by the PCs who checked whether both the participant began and ended the coaching session. If either the participants or CHW did not join, the PC could join the call to help the attendee. Feedback was obtained from the participant about the coaching session through emails after the session and by inviting participants to a follow-up interview after the intervention (see qualitative evaluation).

### 2.7 Evaluation

The evaluation was underpinned by the Reach, Effectiveness, Adoption, Implementation, and Maintenance (RE-AIM) framework (31) which allows for an understanding of the multifaceted and interactive effects of personal, social, and environmental factors that determine behaviour; and for identifying behavioural and organisational leverage points and intermediaries for health promotion within organisations and communities. RE-AIM has been used to evaluate programs and setting in public health and community settings and is thought to be particularly useful when evaluate interventions in “real-world” settings (40, 41). It has also been used to evaluate public health interventions which make use of community health workers in community-based setting (42–44). Results are made up of quantitative measures from the participant questionnaires, qualitative interviews with the participants, the CHWs, VCSE partners, and the research team. Primary quantitative outcome measures included implementation measures such as uptake and engagement and the pre/post changes to the self-report CVD behavioural questions which included the following three questionnaires: (1) the INTERHEART CVD risk questionnaire collected during the screening process was used as the baseline and collected again after completion of the intervention. (2) Physical activity levels were measured using the International Physical Activity Questionnaire (IPAQ) (45). The IPAQ is an internationally validated instrument to capture information about weekly physical activity habits, behaviours, and routines. (3) Diet was assessed using a 20-item questionnaire based on a modified version of the UK Diet and Diabetes Questionnaire (46), a brief food frequency questionnaire designed to assess conformity to healthy eating guidelines, and to assist in the setting of dietary goals. It was used to estimate the number of portions eaten daily or weekly of fruit and vegetables, oily fish (or alternative), and foods high in fat, salt, and sugar, what proportion of the time wholegrain cereal products were chosen, weekly units of alcohol consumed and the frequency of binge drinking. Due to the small sample sizes and non-parametric data used in this study, Wilcoxon Sign test was used to evaluate for differences in continuous variables whilst McNemar's test was used for binary categorical data. The pre-intervention assessment of the primary outcome measures was sent to participants before they participated in the intervention (no participant could begin the intervention without completing the baseline measures). Post intervention primary outcome measures were collected after their participant in the intervention was completed.

Focus groups and one-to-one interviews were conducted with four groups of stakeholders: (1) VCSE partners; (2) CHWs; (3) members of the research team, (4) participants in the intervention. Individual interviews were conducted with VCs, members of the research team, and participants, while data from the CHWs was collected in focus groups. Discussion guides for VCs, CHWs and members of the research team all included questions on the respondents' role within the project, the process of community engagement, barriers, and facilitators the implementation process, recommendations for the future and sustainability. Discussion guides for participant interviews included questions on how and why participants became involved in the project, their experience of the health coaching, and their views on the impact and usefulness of the project. Interviews and focus groups were conducted online using Zoom or MS Teams. The analysis was conducted by TGJ, IR, and RD and using qualitative framework analysis based on the components of the RE-AIM framework. Following data collection interviews and focus groups were transcribed by a professional transcription service and TGJ, IR, and RD familiarised themselves with the full set of data. They then undertook line-by-line coding of the data in NVivo using descriptive primary codes which were then interlinked with secondary codes. These secondary codes were then organised under the five elements of the RE-AIM framework (Reach, Effectiveness, Adoption, Implementation, Maintenance). The analysis was interpreted, findings were synthesised with reference to the stakeholder group and theme descriptions were produced with supplementary illustrative quotes.

The Reach of the intervention was assessed through recruitment rates for the VCSE partners, CHWs and Intervention participants and qualitative data collected from the VCSE partners, and the research team was used to understand barriers and facilitators to recruitment. Effectiveness was assessed during the primary outcome measures and barriers and facilitators to effectiveness were assessed through qualitative interviews with the participants and CHWs. Adoption was at the setting level was determined through assessment of the retention of VCSE partners and qualitatively through interviews with VCSE partners and the research team. At the individual level, Adoption was assessed through CHW retention rates and qualitatively assessed through interviews with the research team and the CHWs. Implementation was assessed qualitatively through interviews with the intervention participants focusing on intervention fidelity. Maintenance was assessed at the setting level qualitatively through interviews with VCSE partners and the research team and through a report of the status of the intervention after 6 months. No individual level maintenance data is reported. A description of the data sources which contributed to each component of the RE-AIM framework is listed in Table 1.

**Table 1:** Description of data source used to evaluate the SPICES-Sussex intervention for each of the RE-AIM components.

Dimension	Data Source	Description of the data source
REACH—The number of VCSE partners, CHWs and intervention participants and the facilitators and barriers to reach and recruitment to the intervention	Recruitment of VCSE partners	Recruitment and engagement of VCSE partner organisations within each research site.
	Community health worker Recruitment	This covered the number of CHWs recruitment and trained by the project and the number who went on to deliver the intervention.
	Participant recruitment	This included uptake of the intervention by eligible participants who completed the screening questionnaire. Only individuals classified as being at “Moderate risk” of CVD were eligible for the intervention.
	Reasons for non-participation	An online questionnaire of eligible participants who did not agreed to take part in the intervention. This questionnaire aimed to understand why eligible participants did not take-up the intervention.
	Facilitators and barriers to Recruitment	One-to-one interviews with VCSE partners and intervention participants. The topic guide asked questions about the views and experience of participant recruitment and the screening questionnaire. (Table 3)
EFFECTIVENESS—The impact of an intervention on primary outcome measures and facilitators and barriers to the intervention for participants.	Primary outcome measures	Quantitative baseline datae and post intervention data from the INTERHEART, IPAQ, and UKDDQ. (Figure 1, Supplementary Appendix 1)
	Facilitators and barriers of intervention effectiveness	Qualitative data comes from the interview with intervention participants. It covered their views and experiences of which intervention elements acted as barriers and facilitators to their experiences of the intervention (Table 4).
ADOPTION—The number, proportion, and representativeness of settings and CHWs. Facilitators and barriers to VCSE partnership (setting level) and CHW experience of the intervention (individual level).	Retention of VCSE partnerships (setting level)	Quantitative data showing the number of VCSE partner sites retained through the intervention and reasons for withdrawal.
	Facilitators of VCSE partnerships (setting level)	Qualitative data discussing the factors that impacted VCSE retention with the intervention (Table 5)
	Retention of CHWs (individual level)	Quantitative data showing the number of CHWs retained through the intervention.
	CHW training needs feedback (individual level)	Questionnaire data with CHW feedback on the training provided by in advance of the intervention.
	CHW facilitators and barriers to adoption of the intervention (individual level)	Qualitative data discussing the facilitators and barriers to the CHW experience of the intervention. (Table 6)
IMPLEMENTATION—Fidelity and consistency to the various elements of the intervention and facilitators and barriers to fidelity and consistency.	Participant retention and fidelity	Quantitative data showing retention and fidelity for intervention participants.
	Participant facilitators and barriers to implementation	Qualitative data discussing the facilitators and barriers to the participant experience of the intervention. (Table 7)
MAINTENANCE—The extent to which the intervention become part of the VCSE partner's practices and facilitators and barriers to sustainability.	Status of the intervention	An update on the status of the intervention 6 months after study closure.
	Facilitators and barriers of intervention effectiveness	Qualitative data comes from the interview with VCSE partners and members of the research team. It covered their views and experiences of which intervention elements acted as barriers and facilitators to maintenance of the intervention (Table 8).

### 2.8 Ethics

Ethical approval for this research was obtained from Brighton and Sussex Medical School's Research Governance and Ethics Committee (R-GEC) (application reference: ER/DG241/17BSMS9E3G/1). This ethics review covered the methods described herein, key research materials, and recruitment and consent protocols for both intervention participants and staff/CHW interviews. Due to the changes imposed on the project by COVID-19 (see Supplementary Appendix 2) and because of minor adaptations from research site to research sites; several minor amendments were made (final application reference: ER/BSMS9E3G/6).

Informed consent was obtained in three ways from study participants depending on the nature of their participation. (1) Online screening questionnaire: these participants were presented with an approved information sheet on the first page of the online screening questionnaire, they were then provided with an Informed Consent Form (ICF) which they had to sign with a digital signature. (2) Intervention participants: just prior to participation and data collection participants met with a research staff member to review the information sheet and to sign the ICF if they agreed to participate, consent was sought again for those intervention participants who took part in a post-intervention interview. (3) CHW and research staff members: participants were sent the information sheet and consent form several days before their interview and were asked to sign and return the ICF prior to their interview appointment.

## 3 Findings

### 3.1 Participant characteristics

Risk profiling data was collected from 381 participants (Females: 310, Males: 71; mean (SD) age = 58 (12.39) years. Forty-Six participants began the intervention (39 Females, 7 Males; age = 58 (11.94) years. Sixteen participants took part in one-to-one interviews at the end of the intervention (thirteen females and two males, aged 32–67 years). Seven members of the research team (6 females, 1 male), and four VCSE partners (3 females and 1 male) took part in the research team interviews. Four focus groups with a total of thirteen participants (10 females and 3 males) were conducted with CHWs from each of the research sites. Thirteen participants (no gender data collected) took part in the post-intervention questionnaire for non-participants.

### 3.2 Analytical framework

The remainder of these findings are organised into RE-AIM dimensions with various quantitative and qualitative methods used to evidence each dimension, see Table 1 for a description of each of the data sources. Table 2 summarises concordance and discordance with expectations of the intervention [as described in the study protocol (29)] in line with the RE-AIM framework. Supplementary Appendix 3 summarises changes to the study design from the published study protocol*.* Throughout this section participant codes are used to attribute quotations and references to specific terminology to a respondent. The codes identify the respondent as either a member of the Research team (RT), VCSE partners (VCSE), Community Health Worker (CHW) or Participants (PP). For VCSEs, CHWs and PPs references to their sites are also made (EB, HA, NH, HG). All codes refer to gender and (F/M), and their number within each respondent category.

**Table 2:** Description of concordance/discordance with our pre-implementation expectations of the SPICES intervention for each of the RE-AIM components (29).

Dimension	Expectation	Assessment of Concordance or Divergence with Reasons
REACH	The majority (>50%) of participants to be in the target demographic, i.e., IMD based on address in 3 most deprived deciles	30% of participants had an address with a postcode in IMD deciles 1–3, below the target figure of 50%. Planned face-to-face recruitment activities which would have taken place within the target areas could not take place as a result of the COVID pandemic and recruitment relied instead on social media adverts. As a result of low response to these adverts the recruitment area was expanded to include more affluent adjacent areas.
	A sample size of 100–150 participants to be recruited to take part in the intervention	Forty-six participants took part in the coaching intervention. The number of participants recruited was lower than expected, recruitment strategies were impacted by the COVID pandemic (see above).
	Roughly equal proportions of men and women to be recruited and to complete the intervention.	Substantially more women than men were recruited—the proportion of women in those recruited to and completing the intervention were 89% (41/46) and 87% (26/30) respectively. This is similar to the proportions of women and men engaging with the social media adverts for the intervention (80% women). Modifications to the intervention should be considered to increase its appeal to men, potentially by utilizing “men-friendly” spaces and including activity-based and group sessions. While some of these activities were considered in the planning stage of the interventions, restrictions due to the COVID pandemic resulted in the intervention being delivered virtually.
EFFECTIVENESS	Coaching programme improve lifestyle risk factor behaviours amongst participants, i.e., improve diet, increase exercise, reduce smoking, control and drinking.	Significant changes were observed in the majority of the target behaviours, with reductions in physical inactivity, increases in fruit and vegetable and wholegrain intake and reductions in intakes of sugary, salty and fatty foods. There was no change in smoking prevalence, but this was very low both before and after the intervention (4%)*.
	The intervention is acceptable to participants	Participant interviews overall suggested favourable views of the intervention, with positive aspects including supportive and non-judgemental coaching, personalised support, and a feeling of empowerment. Negative aspects included being given generic or inappropriate advice and a “lack of direction” in some coaching sessions.
ADOPTION	At least half of the organisations who were engaged with during the pre-implementation phase then carried out the project	50% (four out of eight) of the VCSEs approached delivered the coaching programme.
		Reasons for not adopting the intervention including disruption caused by the COVID pandemic, availability of funding, staff recruitment issues, and the intervention being a poor fit with the organisations' main remit.
	Approximately five volunteer CHWs to be recruited per site and trained at each research site	The number of volunteers recruited per site and completing all training sessions ranged from 5 to 9. Volunteer recruitment was facilitated by local contacts and existing volunteer pools at the partner VCSEs.
IMPLEMENTATION	At least half of participants to complete the intervention by attending a minimum of three coaching sessions.	63% (29/46) of participants completed at least 3 sessions, and 59% (27/46) completed all 6. Attrition did not exceed the anticipated value of up to 50%. Dedicated administrative support was key to scheduling appointments, as rates of cancellations and rebooking by both participants and volunteers were high.
	Coaching sessions to be delivered at approximately monthly intervals.	The median (IQR) interval between coaching sessions was 28 (22, 35 days). Some participants enrolling at sites recruited later in the SPICES programme had sessions more frequently than monthly in order to complete the 6 sessions before the programme ended.
	Co-design process leads to in useful local adaptations to the coaching programme.	The local co-design process included discussion of recruitment methods, the form of the intervention, and the CHW training programme and “charter”. This process worked well for sites joining at the beginning of the SPICES programme. However, CHWs sometimes seemed unsure of their role in this, and the capacity for meaningful adaptation was less for sites joining later in the programme when delivery methods became more fixed.
MAINTENANCE	Changes to behavioural risk factors are maintained for at least 6 months after the coaching ends.	Due to delays in starting the SPICES programme resulting from the COVID pandemic restrictions it was not possible to follow-up the participants for long enough to assess this.
	At least one VCSE to continue the coaching after the end of the SPICES programme	One partner VCSE organisation continued the coaching intervention beyond the lifetime of the programme. This partner recruited a team to implement the programme and retained many of the same CHWs. Funding was supplied by an UK national health service grant.

* Target behaviours were Reduce/cease smoking, increase moderate physical activity, reduce fat, salt, and sugar content of the diet, increase intake of fibre, fruit and vegetables and oily fish (or alternatives), reduce sedentary hours.

## 4 Reach

### 4.1 Recruitment of voluntary and community sector enterprise partners

A community-mapping exercise was carried out during the pre-implementation phase of the project (30) in which three partner organisations were identified across three research sites in East Sussex (Hastings, East Brighton, and Newhaven). All these organisations were volunteer based community organisations with a focus on local community development and improving health, with the Hastings organisations being focused on health and wellbeing. During the intervention set up phase, the East Brighton organisations dropped out of the study due to the impact of Covid-19 whilst the Hastings, and Newhaven organisations were carried forward to deliver the intervention. The East Brighton organisations helped the research team to develop links with a health and wellbeing organisation that was associated with a local General Practice (GP) clinic in East Brighton. Finally, a fourth research site was identified in West Hove and a final VCSE partner was identified. This organisation was a local community development organisation for the area. In total four VCSE organisations were partnered with across four research sites. In each site a VC was recruited from the partner organisation to deliver the intervention with the research team.

### 4.2 Community health worker recruitment

The research team and VCSE partners recruited 38 individuals who attended the introductory CHW meetings (Gender: 27 females and 11 males, NH *n* = 7, EB *n* = 13, HG *n* = 10, HA *n* = 8). Twenty-seven of these individuals completed the full training for CHWs (20 females and 7 males; NH *n* = 5, EB *n* = 9, HG *n* = 7, HA *n* = 6).

### 4.3 Participant recruitment

Social media recruitment had a wider reach to potential participants compared with gatekeeper recruitment, however, several participants did not complete the REDCap screening questions, had a poor understanding of the study, or were not part of the study's target population. VCSE gatekeepers yielded poor recruitment results apart from when a newsletter with a particularly large reach was used. Social media was the primary strategy for recruiting participants to the study. In total the messages reached 13,086 individuals across four waves of recruitment and of these 472 (3.6%) engaged with post by clicking on the survey link. Of those who clicked on the link 80% were female and 20% were male.

**Figure 1:**
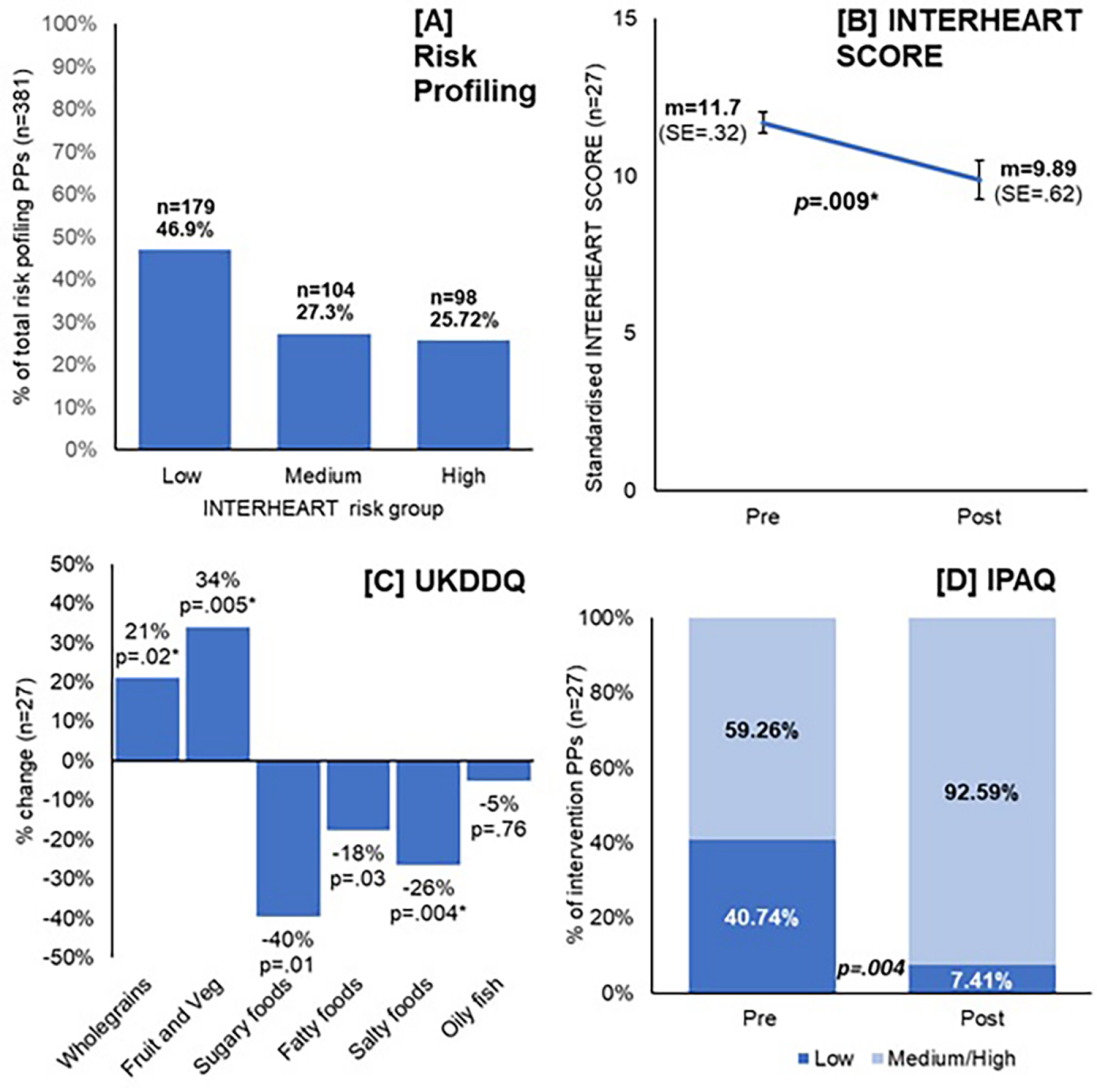
Primary outcome measures for the “Reach” and “Effectiveness” components of the SPICES-Sussex intervention. (**A**) The proportion of “Low”, “Medium”, and “High” risk participants identified during the Interheart risk profiling questionnaire; (**B**) the mean Interheart score pre and post intervention for those who completed the intervention, *p* value from paired *t*-tests; (**C**) shows the % change regularly of dietary behaviours from pre/post intervention UKDDQ score, within-group *t*-tests; (**D**) the change in the % of intervention participants classified as having either low or medium/high activity levels pre and post intervention, *p* value from McNemar's test.

The INTERHEART screening data is shown in Figure 1 and Supplementary Appendix 1 for all those who completed the screening questionnaire (*N* = 381), participants who started the intervention and then withdrew (*N* = 17), and participants who completed the intervention and on whom we have full data (*n* = 27). Of the CVD risk factors measured by the INTERHEART screening tool, the two most prevalent were stress (reported by 61% of those screened, 56% of those who started the intervention, and 78% of those who went on to complete), and physical inactivity (reported by 55%, 81% and 64% respectively).

Forty-six participants took part in the CVD coaching intervention across the four research sites, all of whom completed the pre-intervention quantitative questionnaires. Sixty-three percent completed the full coaching intervention, and one participant withdrew from the project after three months. We had full data for twenty-seven of twenty-nine participants who completed the full 6-month coaching intervention (note: these participants have been removed from Supplementary Appendix 1, *n* = 2), Participants' characteristics are summarised in Table 3. Several participants withdrew (37%), reasons given for withdrawing were: ill health/poor mental health/ill health in the family (13%); the intervention was considered a poor fit for the participant/did not meet their expectations/they did not need the intervention (9%); other commitments got in the way/they were too busy with their normal lives (7%); repeated non-attendance at planned coaching sessions from the CHW (4%); did not get on well with CHW (2%), language issues (2%).

**Table 3:** Facilitators and barriers to reach and recruitment.

Name	Description	Illustrative quote
Experience of CHW recruitment	CHW recruitment was generally considered to be a success by the research team and the VCSE partners. This was largely due to the partnership between the University and the VCSE organisations, as these partners were able to leverage social capital and trust in order to mobilise their existing community and volunteer base.	*“I think the people that responded were the ones that worked with us before and trusted us, they knew they were going to probably have a positive experience volunteering on a new project.” (RT-F-02)*
	Following a difficult experience with one CHW, a one-to-one CHW induction session was added at the second and subsequent study sites prior to the training. This aimed to assess CHW personal qualities, motivation, and suitability for the intervention.	*“I think we were so excited to take on volunteers and get them into the project that we were happy to take anyone and train them up but … we know now that we also need to interview them and make sure they are right for the project going forward.” (VCSE-HAF-01)*
Community partnerships	The perceived benefits of recruiting in partnership with VCSEs was mentioned by CHWs and the research team. It was thought that links to recognised community organisations would improve trust and allow for access to VCSE community networks.	*“…they don't know us, whereas there's a community group in the community they know… it's real, it's tangible… so I think going through community groups was the best way” (RTF-06)*
	However, despite VCSE partners being perceived as a positive for reach into the community, members of the research team noted that this did not seem to play out in practice, and that whilst VCSEs were effective in recruiting CHWs, they had not had the anticipated reach to recruit participants.	*“…we thought they would have the reach in the communities to do participant recruitment, but they just didn't”. (RTF-02)*
	Participants stated that they took part in the intervention because they were “open-minded” to opportunities (NHF-02) or that the intervention came at the right time for them (HAF-04). Additionally, the free coaching was a major facilitator for some participants, especially for those on a low income. Other factors raised by respondents included the helpful nature of personalised emails in building a rapport with potential participants and facilitating recruitment.	
The risk profiling questionnaire	The online INTERHEART screening tool was felt to present barriers to recruitment by all the VCSEs and several members of the research team. These included what was perceived to be its unnecessarily complicated nature, technical difficulties in completing it, and possible digital exclusion in using it with marginalised people. There was a mixed view of the risk profiling tool amongst study participants. Participants understood why they were being asked the questions and felt that for the most part they were clear and easy to answer (PP-NHF-01). Some mentioned that the questionnaire was too simplistic to capture their experience, and the requirement for a tape measure for waist and hip measurement was said to be a barrier. Many found the REDCap platform somewhat “clunky”, “tedious”; or annoying to use and that it was reductionist or irrelevant to their own personal experience of their health (PP-HAF-09, PP-HGF-11).	*“I found that a bit clunky. It was the clicking of the options and then trying to select and the whole journey, and then it felt quite tedious and long to get through.” (PP-NHM-09)*
Impacts of COVID-10	Restrictions resulting from the COVID pandemic presented several barriers to participant recruitment. One of these was the inability to promote the intervention with face-to-face community engagement due to COVID restrictions, with this point being raised by volunteer co-ordinators, CHWs and members of the research team.	*“We've done emails to people to promote it as much as we can really on our side without overdoing it […] if we weren't in COVID times […] then obviously we would be going out physically and promoting the project”. (CHW-RTF-16)*

Due to low initial recruitment rates, the recruitment areas were expanded and included more affluent adjacent areas. The proportion of those completing the screening questionnaire and of those who went on to start the intervention who were in the target population (i.e., had an address with a postcode and IMD in the most deprived three deciles) was 30% in both cases. Despite recruitment being gender neutral and without gender/sex related parameters on social media our risk profiling questionnaire recruited far more women than men (77% female, 23% male, see Supplementary Appendix 1). This issue was carried forward to the main intervention in which only five of the forty-six who initially took part in the study were male.

### 4.4 Reasons for non-participation

Reasons given for not participating included missing or not receiving an invitation to take part (*n* = 4), lack of time due to responsibilities and commitments (*n* = 4), not feeling like the intervention was a good fit for them and their circumstances (*n* = 2), not being happy with the CHW allocated to them (*n* = 2), being reluctant to take part in online activities due to a lack of privacy at home (*n* = 1). When asked what would have made them more likely to participate the most common response was more clarity/detail on what was involved (*n* = 3).

### 4.5 Facilitators and barriers to reach

Intervention participants referred to several intervention components that functioned as facilitators or barriers to the reach of the intervention. These barriers and facilitators were organised into themes which include: (1) Experience of CHW recruitment; (2) The value of community partnerships; (3) The experience of the risk profiling questionnaire; (4) Impacts of COVID-19. These barriers and facilitators are described in more detail in Table 3 and illustrative quotes are provided.

## 5 Effectiveness

### 5.1 Primary outcomes measures

For those participants who completed the intervention, the before and after measures of cardiovascular risk, diet, physical activity, and readiness to change were compared (see Figure 1 and Supplementary Appendix 1). Mean INTERHEART score fell significantly from 11.7 to 9.9, taking the mean to within the low-risk range. There were also significant improvements in the self-reported dietary measures including: an increase in the proportion of time wholegrain foods were chosen, and the daily portions of fruit and vegetables eaten, and decreases in the consumption of fatty, salty, and sugary food. No changes were observed in the consumption of oily fish. Self-reported levels of physical inactivity also dropped over the course of the intervention with the proportion of those classified in the “low” physical activity category falling from 40% to 7%. Additionally, the self-reported levels of participants' “readiness to change” during the intervention increased from 3.6 to 4.5, which indicates increased levels of motivation as a result of the intervention.

### 5.2 Participant reported facilitators and barriers to the effectiveness

Intervention participants referred to several intervention components that functioned as facilitators or barriers to the effectiveness of the intervention. These barriers and facilitators were organised into themes which include: (1) accountability—the ways CHWs kept participants accountable about their health behaviours; (2) connection and community—the importance of making human connections with the CHWs and feelings of community togetherness; (3) judgement-free—the importance of a judgement-free intervention experience; (4) motivation and support—the coaching role that the intervention took in the lives of participants; (5) personalisation—the feeling that the intervention was adapted to their own needs and experiences; (6) reflection—the value of reflecting on experiences during the coaching intervention; (7) self-efficacy—the ways in which CHWs made participants feel in control of their health behaviours; (8) gradual or modest impact—the feeling that the intervention largely lead to modest impacts (9) generic or inappropriate advice—the feeling that the information provided during the coaching was too generic, obvious, or inappropriate to their needs. These barriers and facilitators are described in more detail in Table 4 and illustrative quotes are provided.

**Table 4:** Facilitators and barriers to intervention effectiveness.

Name	Description	Illustrative quote
Accountability	CHWs acted as a way of keeping participants accountable to the behaviour or life changes. The regular appointments meant that the behaviour change was “always at the back of my mind” (PP-HAF-03). and that participants would think about “feeding back” their progress to throughout the month (PP-HAF-09).	*“…it would have been embarrassing to go back at the end of the month and telling them I'd put on weight.”* (PP-NHF-01)
Connection and Community	Being able to talk to someone regularly and openly about their health was seen as a positive of the intervention. For some, the fact that the CHW was a stranger facilitated this openness (PP_EDF-14).	*“…she was able to get down to you know, where I was at and what I needed.” (PP-HGF-11)*
	For participants who struggled during the COVID-19 restrictions, who were retired, or who had busy working lives the connection provided by the CHWs combated loneliness that they often experienced. The knowledge of the local community was useful when CHWs had it or when the participant and the CHW shared interests or background, however it was often not that important to participants (PP-HAF-04).	
Judgement-free	CHWs generally treated participants without judgement of their lifestyles. Rather than being “draconian” (PP-HGM-12) or being made to feel “stupid” or “wrong” the CHWs provided a safe space in which participants could discuss “options” to change their behaviour (PP-HGF-06). Some participants reported feeling judged, however, and explained this was a result of the nature of the intervention itself, which focused on the need to change their behaviour, rather than of feeling any judgement from their CHW specifically.	*“The reason I haven't been [to lifestyle management interventions] before, is because I thought they were all going to be, erm, quite draconian in the way that they delivered their method. But it [the intervention] wasn't like that at all.”* (HGM-12)
Motivation and Support	The feeling of being supported and mentored by the CHWs was one of the key draws. For some, this support took the form of emotional “encouragement, friendliness and a sense of humour” (PP-HGF-11) and for others the motivation took the form of a “push in the right direction” (PP-HGM-12).	*“…to my friends I've said that she's [CHW name] like a mentor. That [CHW name] was a mentor to me.”* (PP-HGF-11)
	Participants often reported that the CHWs made them feel as if they had been too hard on themselves during their past, or that they had felt like a failure in the past (EBF-05). This softer form of motivation and support was appreciated by participants.	
	Participants generally had good knowledge of CVD health risk factors and they knew that their own health was poor and that they needed to make lifestyle changes. However, they reported feeling as though they needed extra support and motivation to push them to make changes.	
Personalisation	Participants reported that the one-to-one coaching felt that it was personal to them or linked back to their “story” (PP-NHF-02). This was facilitated by the feeling that their CHWs were listening intently to what they said and incorporating it into their personal behaviour change journey (PP-NHM-09), and “going the extra mile” for the participants.	*“She went the extra mile, every time we finished a session, we'd recap. And then the following session, she'd come back, and she'd done a bit of research for me, which I wasn't expecting her to do.”* (PP-NHF-02)
Reflection	Reflection on past sessions was used as a tool used by CHWs to promote and document the participant's behaviour change. CHWs used past behaviours as a benchmark for their progress or reflected over their achievements at the end (PP-NHF-02). Both participants and CHWs made use of notetaking which helped to facilitate this reflection (PP-NHM-07).	
Self-efficacy	Rather than giving commands or specific instructions to participants CHWs would often work alongside the participants as equals (PP-HAF-03). This allowed participants to feel more “in control” (PP-NHF-02) and more empowered around their health, and as they advanced, participants reported feeling as though they had learnt to respect themselves (PP-NHF-15).	
Gradual and modest impacts	Initial expectation amongst participants was that they had to make drastic lifestyle changes to improve their health. However, participants came to realise that minor, achievable, and gradual changes could be impactful and should be celebrated (PP-HGM-12).	*“I do feel a little bit proud of…because I have lost a little bit of weight, I'd like to lose a bit more, erm, but it's only been through sort of small changes.” (PP-HAF-03)*
Generic or inappropriate advice	Participants often reported that the coaching sessions lacked purpose or direction and that sometimes it was “difficult to know” what they were trying to achieve (PP-NHF-01). The health advice given to them was sometimes reported to be inappropriate to their specific needs (i.e., health conditions) or too basic, obvious to the participant, or unhelpful.	*“It was difficult to know what we was [sic] trying to achieve I think sometimes. … we had a couple of times where we waffled round in circles and didn't really get anywhere.” (PP-NHF-01)*

## 6 Adoption

### 6.1 Retention of voluntary and community sector enterprise partnerships (setting level)

Of the six VCSE organisation engaged with during the pre-implementation phase of the project, four went on to be VCSE partner organisations during the implementation phase. Disruption and staff pressures resulting from the COVID-19 pandemic were a significant barrier to recruiting partner VCSEs, with two organisations who had been involved in initial discussions deciding not to proceed for this reason. Furthermore, interruptions to communication caused by COVID-19 and research team changes led to a loss of trust and engagement in some cases. One organisation which had a group of people ready to volunteer at the beginning of the project later withdrew as this group had fragmented due to COVID-19-related delays and substantial staffing changes that took place just prior to the implementation phase between 2019 and 2020. Other factors impacting on VCSE recruitment included the availability of funding, and issues with recruiting staff to the VC role. After one of the VCSE partners dropped out of the study just prior to the implementation phase, the same organisation linked the research team with another organisation who eventually functioned as VCSE partners for the implementation phase. The need to develop trust, and having the time to achieve this, was stated by several members of the research team as being key to recruiting partner VCSEs. Quality of communication was also felt to be especially important.

### 6.2 Facilitators of voluntary and community sector enterprise partnerships (setting level)

VCSES and research team members referred to several intervention components that functioned as facilitators or barriers to setting level adoption. These barriers and facilitators were organised into themes which include: (1) Trust—the importance of developing trust with community- partners; (2) Local Knowledge—the value of local knowledge and to delivering appropriate community care; (3) Local Skills—the value of the skills and experiences in local communities to delivering the intervention. These barriers and facilitators are described in more detail in Table 5 and illustrative quotes are provided.

**Table 5:** Facilitators and barriers to intervention setting level adoption.

Name	Description	Illustrative quote
Trust	The need to develop trust and having the time to achieve this was stated by several members of the research team as being key to recruiting partner VCSEs. Quality of communication was also felt to be very important.	*“It needs time for them to see you and understand that you mean well, and you'll be around when something goes wrong, that's the most important thing… It's basically about listening skills and not going in and assuming you have all the answers.” (RT-M-03)*
	A key advantage of using the voluntary sector raised by VCs, CHWs and members of the research team, was that it was felt to promote trust in the strategy to implement the intervention and help it to feel embedded in the local community.	
Acceptability of CHWs	CHWs were also felt to have useful local knowledge, and to be “on a similar level” to participants, helping to reduce intimidation and the fear of judgement when talking about health and lifestyle.	*“I think a volunteer is maybe closer to the people in the community than somebody who's an official, you know, maybe a doctor… I've found that the ladies that I'm coaching are very happy, and they don't feel intimidated… they feel almost like we're two friends and we're going on this journey together.” (VCSE-HAF-01)*
Use of volunteers as CHWs	Several respondents also pointed out that CHWs could be a good way of dealing with workforce shortages and did not need to be paid. Conversely, the CHWs lack of experience and contractual obligation was felt to result in several disadvantages. These included frequent cancellations of coaching sessions due to competing priorities such as work or childcare, and some CHWs were felt by the VCs to lack commitment. They were also felt to sometimes lack confidence in dealing with complex issues.	*“The whole way from the very beginning we told them you're not an expert, you're not a health professional, you don't have to have all the answers and yet it still contributed to them feeling like not particularly confident sometimes.” (RT-F-07)*
	One VC commented on the high level of investment required from the research team to achieve this (to note, an economic assessment of the project was not made).	*“it feels like a sort of a lot of investment, lot of capacity on … the sponsor side to make that happen … how sustainable that is I don't know.” (VCSE-EBM-04)*

### 6.3 Retention of community health workers (individual level)

Of the twenty-seven CHWs who were recruited and trained to be a part of the intervention, twenty-one went on to deliver one or more session as an active CHW (Gender: 15 females and 6 males NH *n* = 5, EB *n* = 6 HG *n* = 5, HA *n* = 5). Each of these CHWs completed the intervention with at least one participant and the maximum number of participants who completed the intervention with one CHW was three.

### 6.4 Community health worker training needs feedback (individual level)

After training sessions in our first site, a short questionnaire was conducted with CHWs who attended the training in the formof one-to-one discussions with the training coordinator and the research team. Questions were asked about the anticipated barriers that CHWs thought they would face during the coaching as well as key training needs. Anticipated barriers and challenges during the project included: a sense of mistrust amongst participants, issues of poverty and deprivation, triggers, and sensitivities to the experiences of participants (i.e., trauma or addition triggers). The key training needs identified included: the sharing of personal stories to empower participants, how to set achievable health goals, preparing CHWs with tools to challenge the participant in a supportive way, improving CHW confidence, and advice on how to communicate CVD risk to participants in a straightforward way.

### 6.5 Community health worker facilitators and barriers to adoption (individual level)

CHWs referred to several intervention components that functioned as facilitators or barriers to the adoption of the intervention at the individual CHW level. These barriers and facilitators were organised into themes which include: (1) Local adaptation and Codesign Sessions; (2) CHW motivation for participating; (3) CHW experiences of the training; (4) CHW experience of the support provided to them. These barriers and facilitators are described in more detail in Table 6 and illustrative quotes are provided.

**Table 6:** Facilitators and barriers to intervention individual level adoption.

Name	Description	Illustrative quote
CHW experience of local adaptation and co-design	Several topics were discussed during the local adaptation sessions. These included the name to be used for the project at each site, methods of participant recruitment, the form of the intervention experience for CHWs and participants, the training programme, and the “volunteer charter”. VCs and CHWs generally appreciated this process and commented that they felt included in decision making and that their input was valued.	*“I think that's been … kind of a strength of like the project is the collaborative nature of being open to hearing different viewpoints and ways of working and thinking, right, how can we accommodate and how can we put this together into a shared plan.” (VCSE-HGF-02)*
	However, members of the research team commented that while the adaptation sessions worked well for the first sites, there was less room for local adaptation at the later sites to join, both because methods of delivery became more fixed as the study progressed, and because of a lack of time to implement changes. They also commented that CHWs sometimes seemed unsure what was expected of them in the co-design process.	*“it worked really well for the first site … and then I think it became a little bit more challenging later on because we were going to them kind of saying, oh, we want to hear your ideas, but actually this is kind of already set.” (RT-M-01)*
	The local adaptation led to some changes in the way the study was rolled out between the sites. These were mostly focused on the support offered to the CHWs, including regularity and format of support meetings and training offered, and recruitment and outreach approaches, rather than changes to the design of the intervention or participant experience.	*“it was almost like they were asking us to come somewhere and get asked a bunch of questions about a programme that they don't know about.” (RT-F-02)*
Motivation for CHW participation	Many of the CHWs had a background in health-related areas, such as nursing, health coaching or activity and fitness, several also had some experience in counselling and behaviour change. A number stated that their motivation for volunteering was related to experience of their own or a relative's health problems.	*“As a health coach I know that you can change massively your health and take control of it by knowing how to change your habits… I think it's really important because my mother suffers from a heart problem.” (CHW-HAF-01)*
	The desire to “give back” to the community was also mentioned by several CHWs. Some also commented that being involved had helped their own physical and mental health.	*“…my major thing for me in my life is giving back…I went into it basically for that and to help me… it's helped my mental health to be involved.” (CHW-EBF-02)*
	One CHW stated that the CHW role had provided her with potentially useful work experience.	
	Participants showed appreciation for the CHWs, especially when they realised that they were providing their time in a voluntary capacity (HAF-04). However, they questioned what the CHWs were getting out of the project. Some also showed discontent at the fact that the CHWs were not paid.	*“I think I'm going to explore health coach roles after my Masters … so it's given me some really worthwhile experience. “ (CHW-EBF-02)*
	They also often referred to how much they enjoyed the sessions with their CHWs and how they found them to be “pleasant” (HGF-06), “personable” (NHM-06), and “supportive” (EBF-05).	*“I'm just so grateful, honestly, I just can't believe this has been for nothing, it blows my mind, err, because it's really changing my life um for the better.” (CHW-NHF-15)*
Experience of CHW Training	As a result of COVID restrictions the training was online, and two of the VCs commented that it would have been better delivered face-to-face as originally planned. Comments from VCs and CHWs on the training programme were generally positive although several VCs and CHWs felt the programme was a bit rushed and intense, and several CHWs commented that they would have liked more time for role play within the sessions.	*“A bit of information overload … I think they found it a bit overwhelming” (CHW-HGF-02)*
		*“I probably didn't use everything … but I just felt confident… the knowledge from the training just gave me the confidence to sort of carry that on”. (CHW-EBF-02)*
	Both CHWs and VCs noted that instilling confidence in coaching skills, rather than just information, had been a key part of the value of the training.	*“I knew she had training in, I don't know exactly what it was, but I knew she had training and she'd done certain things herself, and she was interested in psychology and things like that. And she brought that in, to our conversations, which I found really helpful.” (CHW-NHF-01)*
	Participants described the CHWs as “professional” (HAF-04), “knowledgeable” (HGF-05), and “wise” (NHF-15). CHWs bought in skills of their “background” experiences to the coaching.	
	However, some CHWs showed some signs of inexperience such as nervousness (HAF-09), a lack of practical suggestions (NHF-16), or tendency to talk about themselves (HGF-06).	
Experience of CHW Support	The administrative support provided by the research team was considered as “friendly” (HAF-09), “straightforward” (HAF-04), and “informative” (HGF-11). These roles were essential for ensuring that the participants had a consistent and trustable contact which supported their coaching sessions. In addition, the PCs provided IT support which was frequently requested by CHWs due to difficulties accessing documents or delivering the online coaching. CHWs spoke highly of the administrative support offered.	*“Just, yeah, really supported by the administrative team throughout the whole process. They were really quick to respond.” (CHW-RTF-05)*
		*“there's so many cancellations, we just need to keep on top of everything… we had it kind of doubled up so that if we miss something somewhere… we wanted to have like a duplicate somewhere else.” (RTF-02)*
	However, rates of cancellations and rescheduling of appointments by both CHWs and participants were high, resulting in a considerable workload for the PCs and the need for effective tracking systems.	*“…we almost tailor, you know… oh hello, hope you had a lovely holiday …we're so friendly, and we can have that relationship with them because it's a small tracking system and we do it all ourselves.” (RTF-01)*
	The PCs suggested that for the project to work on a larger scale, auto-correspondence for reminder emails would be needed, but noted that this would result in the loss of personalised messages which they felt helped build relationships with participants.	
	Most CHWs spoke very highly of the support received, which was felt to be particularly important when they were building confidence at the beginning of their coaching role.	*“The support is brilliant; we know that we can contact anyone… and in our meetings every month it's been very useful to share ideas and to share our thoughts.” (HGF-03)*
	Several respondents mentioned that feeling part of a team was an important part of the CHW support.	
	The personal relationship between CHWs and support staff in which a feeling of care, connection, and/or reassurance, was provided was repeatedly raised as an important feature of the overall support package.	*“I think they really benefitted from being brought together for kind of action learning meetings every month and feeling like they were part of a team, I think them having to do it in isolation wouldn't have worked.” (RT-F-04)*
	Several CHWs noted that many of their participants were suffering from stress and had complex emotional and mental health needs, often linked to the COVID pandemic, and the value of support given by the VCs and health coach when dealing with this.	
	CHWs made substantial use of the signposting and resources that the research team prepared for them. They also went beyond these by actively seeking out resources that met the participant's needs and goals. CHW's often also brought their own expertise and knowledge from their lives to the coaching i.e., sharing resources for a physical activity that they participated in (HGM-12).	*“…a couple of the volunteers would almost want to talk before a session and after a session… it was just connection and a chat and feeling listened to and supported.” (RTF-07)*
		*“…the first session I had with one of the participants … she was very emotional and cried… I think [CHW trainer] was surprised that this lady had so much going on … she was concerned that I was taking on a lot from her so it was very helpful to speak to both of them.” (CHW-NHF-01)*

## 7 Implementation

### 7.1 Participant retention and fidelity

Overall, 48% (*n* = 51) of those eligible (*n* = 106) to take part in the intervention agreed to do so and provided consent, of those 90% (*n* = 46) attended their first CHW coaching session and completed the baseline questionnaire. Of those who completed their first session 63% (*n* = 29) completed three sessions and 45% completed six sessions. For the 46 participants that began the intervention there were 276 planned program contacts of which 183 (66%) were completed. Retention and attendance data are summarised in Supplementary Appendix 2. No data was collected on the amount of time each participant spent in their coaching session.

### 7.2 Participant facilitators and barriers to implementation

Intervention participants referred to several intervention components that functioned as facilitators or barriers to the implementation of the intervention. These barriers and facilitators were organised into themes which include: (1) expectations of the coaching intervention, (2) the virtual coaching sessions; (3) holistic and flexible, (4) length of the coaching session, (5) administrative support, (6) past experiences, (6) mental health. These barriers and facilitators are described in more detail in Table 7 and illustrative quotes are provided.

**Table 7:** Facilitators and barriers to intervention implementation**.**

Name	Description	Illustrative quote
Expectation	Despite numerous attempts to inform participants about what was involved, participants reported having a poor understanding of what the program entailed. Many of those subsequently reported being pleasantly surprised at what they got.	*‘When [CHW name] said to me, “What do you want to get out of this?” Basically, I said, “Well, I don't know”* (PP-NHF-16)
	Participants noted that the intervention was not well suited to people living with complex health conditions, disability, or long-COVID. They experienced the intervention as being based on an “individualistic” assumption about lifestyle change which relied on making changes which were not considerate of their conditions (PP-EBF-13). Poverty and vulnerability were also seen as barriers to making many of the lifestyle changes promoted during the intervention.	
Virtual coaching session	Although some CHWs expressed a view that face-to-face coaching sessions would have been better, most felt that the virtual coaching sessions had gone well and had several advantages. Several CHWs suggested that virtual coaching provided a helpful distance between the CHW and the participants, reducing anxiety on both sides.	*“…because it does have that distance there… maybe makes people more at ease to talk about personal things… if I'd been sitting in somebody's room… I don't know whether people would have opened-up as much”. (*PP-*NHF-01)*
	The convenience of online coaching was also felt to be a facilitator to attendance by both CHWs and the VCs.	*“It's brilliant doing it online, it's sort of personal enough to make you feel guilty, but not so personal that you're uncomfortable by it.” (*PP-*NHF-02)*
	It was felt that cancellation rates would have been even higher if participants had needed to travel to attend sessions in person. Disadvantages of online coaching sessions raised by the CHWs included not being able to see the participants' body language, and the difficulty of sharing information and resources with participants.	
	Study participants were also positive about the use of Zoom, reporting that it was less hassle than face-to-face (PP-NHF-02) because they “didn't have to go anywhere” (HGF-06).	
	Some reported experiencing technical problems such a failing internet connections (PP-NHF-01) and there were issues of digital exclusion amongst participants which made the use of Zoom more difficult.	
Holistic and Flexible	The “freedom” and flexibility of the coaching session was seen as a positive(PP-NHF-02, PP-HGF-11) CHWs were not fixed to “one stance” (PP-HGF-06). Sessions were “relaxed” and “laid back” (PP-EBF-09) but they generally fell into a rhythm of discussing progress with previously discussed goals, before having a period of flexible and open conversations, followed by goal setting at the end of the session. Once this rhythm was established participants reported that it did not generally change that much. Rather than being explicitly focused on CVD health, CHWs and participants took a more holistic focus to health. Conversations about one's health would often “open up” into different areas of their lives (PP-HAF-04).	*“it's just that … when you're having conversations it opens up, sometimes it'll just open up your thought processes and send you off in other directions.” (*PP-*HAF-04)*
Length of coaching	Participants felt that 6, monthly sessions were appropriate as this allowed enough time to plan, implement, and experience results of lifestyle changes. Their feedback on the length of individual sessions was mixed, with some stating that they were far too long whilst others found it very easy to fill the hour-long time slot. There was also a desire to carry on with the coaching or to have extra support beyond the initial coaching into a “phase 2”.	*“I think a Phase 2 would be good. … I think a Phase 3 would be good. Our hearts are with us for a lifetime. So, to assess change, it needs to be over a long period of time.” (*PP-*NHF-02)*
Administrative support	The administrative support provided by the research team to participants of the intervention was “friendly” (PP-HAF-09), “straightforward” (PP-HAF-04), and “informative” (PP-HGF-11). These roles were essential for ensuring that the participants had a consistent and trustable contact which supported their coaching sessions.	
Past experiences of care	Negative past experiences of behaviour change were discussed by participants with many reporting that they had made many efforts to change but that they had “fallen off the bandwagon” (PP-NHM-07). Interactions with clinical experts to address lifestyle changes were also commonly discussed and were largely viewed negatively. Firstly, clinicians were regarded as having highly limited time and participants had to repeatedly contact them (PP-NHF-01) or they would meet for very short periods (PP-NHM-07). Secondly, interactions with clinicians were often regarded to be “impersonal” (PP-NHF-01), “patronising” (PP-HAF-09), or “inconsistent” (PP-HAF-09).	*“Well, the clinician's is not personalised at all. it's very automatic. And very clipped and very brief.” (*PP-*NHF-01)*
Mental Health	Despite the focus of the study not being on mental health, participants spoke very openly about their mental health. CHWs validated participants and helped them to realise that they could make changes. Participants also discussed how improving their physical health could feed back on their mental health and improve their mood more generally.	*“…as the meetings progressed, I did say that I was feeling much better. And I was feeling lighter, lighter—not as in weight-wise, but light in spirit.” (*PP-*HGF-10)*

## 8 Maintenance

### 8.1 Status of the intervention after six months

Six months after the intervention's funding period ended the program was being continued at two of the sites. One of the sites continued it as volunteer opportunity and peer support program which was covered by their existing funding for peer support programs. A second site was awarded funding from the National Health Service to continue the intervention. The latter's findings will be reported as a program evaluation in the future.

### 8.2 Facilitators and barriers to maintenance

Interviewees referred to several intervention components that functioned as facilitators or barriers to the maintenance of the intervention at the setting level. These barriers and facilitators were organised into themes which include: (1) continuity of the intervention; (2) funding; (3) infrastructure These barriers and facilitators are described in more detail in Table 8 and illustrative quotes are provided.

**Table 8:** Facilitators and barriers to maintenance.

Name	Description	Illustrative quote
Continuity of the intervention	All the partner organisations indicated that they would be happy to continue with the programme which they felt was a good fit with their other initiatives, if it could be sustainably resourced.	*“Speaking informally to some of the clinicians, they're certainly very keen and it does fit exactly perfectly well with what they're already doing…our community works very well when things are happening from within it, and that's a really important feature for us… I think the fact that … it was done in such a way it could be built almost from within, and that worked really well.” (RT-F-07)*
	They also generally felt that the CHWs would be happy to continue to deliver the programme, but the volunteers' stated views on this in one focus group were mixed—one said they would be happy to continue, another did not want to, and another wanted further training if they were to continue.	
Funding	The main stated requirement for continuing with the intervention was funding, in particular for the VC role, and the possible need to recruit extra staff in order to maintain the project was also mentioned. One VC suggested a small amount of funding for recruitment and promotion would also be required. Several VCs commented that they would need continued support from the SPICES research team to continue to deliver the intervention, particularly with regard to training, recruitment and IT support.	*“it would be nice to be reassured that we've still got you guys to fall back on some of the technical support… and [Health coach name] been just great with the coaching, again that's not my area of expertise.” (NHF-03)*
Infrastructure	Members of the research team noted the need to retain a core support team to deliver the interventions, particularly the health coach and admin roles. Considerable administrative support was required, particularly during participant recruitment. Dealing with booking and rescheduling appointments was extremely time-consuming. The research team also commented that the local primary care system embraced the project much more than was anticipated before its start. Integrating it into that system thus seemed more feasible than we had predicted. We had expected much less enthusiasm from (busy) healthcare providers, so this was really good news.	*“…integrating it into a service rather than just thinking of it as a voluntary sector thing is really good, and seems to be working out well.” (RTM-01)*

## 9 Discussion

SPICES Sussex developed strategies to implement effective community-based CVD risk reduction interventions based on behaviour change coaching with CHWs by partnering with and leveraging the experience and influence of VCSE in four underserved communities in East Sussex, UK. Despite issues with recruitment and challenges associated with COVID-19 as well as other logistic, management, and research design challenges, the project showed clear markers of success. Participants experienced the interventions positively and many made gradual, and sometimes substantial, lifestyle changes. The quantitative results showed significant reduction in participants' CVD risk after taking part in the interventions. We think these successes were due to implementing our interventions in a flexible, personalised, and holistic way, which empowered CHWs to use their skills and experiences to aid participants. These results demonstrate how CHWs-led and community-based preventative CVD interventions could be implemented, such as those seen widely across the Global South (17, 18). They also support a “person-based” and “asset-based” approach to community-based implementation design (23, 25) in which the strengths and assets of communities and their members are used to promote health and wellbeing.

### 9.1 Intervention design

The SPICES-Sussex project used community-engagement and community health worker approaches to improve CVD health that are based on practices developed and tested in Kampala, Uganda (47). As part of the SPICES consortium these practices were adapted to several global north (UK, France, Belgium) and global south settings (South Africa). In the global south social public health approaches have long advocated for the decentralisation of healthcare to community partners and for a greater focus on prevention (48). Community-based public health practices such as task-sharing are often utilised in low-resource health systems in low-and middle income countries by recruiting and training community health workers to deliver low-intensity health intervention such as health coaching and signposting (49). In global south SPICES settings, there was greater buy-in to community-based interventions from governments and much of the trust building, and infrastructure for community health workers already exists (50). These settings, including the SPICES sites that influenced the Sussex site, often rely on voluntary or unpaid volunteers to conduct public health work in order to lower cost and to make use of existing social networks.

In resource-rich global north settings, healthcare is far more institutionalised and focused on secondary care and the infrastructure for community-based and participatory interventions is far less well developed. In the UK, most health interventions must adhere to the institutional demands of the National Health Service which presents a range of resource intensive training, recruitment, safeguarding, and management practices. There is much less history of CHWs in the UK; the role of these workers is not well understood or well defined outside of the third/voluntary sector despite recent calls for their use during the COVID-19 pandemic (51). This squeezed landscape for community-based intervention and the lack of familiarity with the role makes the development and implementation of these interventions challenging. In the global north there are increasing challenges to the volunteer nature of CHWs with researchers calling for compensation, capacity building, or payment of members of the public involved in intervention delivery of research and health interventions on moral and efficacy grounds (52, 53). In our study, the decision not to pay CHW was made as a result of us following the SPICES approach developed in the Global South (17) and because the VCSE organisations we partnered with all had existing unpaid community volunteer programs. In our post-intervention qualitative evaluation interviews, participants and CHWs both discussed the value of paying CHWs. Furthermore, the drop in CHWs and the small number of participants they were able to take on implies that the lack of payment impacted the degree to which CHWs were able to engage with the project and therefore impacted the intervention's effectiveness and sustainability. In the UK, the NIHR now recommends that members of the public who are involved in research are properly reimbursed for their involvement and provide frequently updated guidelines on how to do so (54). In the future we argue that public health intervention that make use of CHWs should reimburse and pay them in some way for their involvement.

Community volunteers with low levels of training (10 h core training plus ongoing support), such as those used during this study, are not well-suited to complex cases or acute needs that required specialised support. In our findings, participants complained of generic, inappropriate, or obvious advice from the CHWs. Participants did not seem to prioritize the knowledge or expertise of the CHWs and instead valued the personalised, holistic, and supportive relationships that were offered by the CHWs. Participants in the intervention reported having good knowledge of what they needed to do to improve their health but struggled to do it in practice. Therefore, this kind of intervention may be well-suited to providing emotional and social support to people at risk of CVD who know what they “should” do but need a support and judgement free support mechanism to make changes.

Interviews with participants revealed a tension in the study linked to the use of an individualistic lifestyle change intervention situated within a community-based and participatory study. The study design did not address community-level, socio-economic, or environmental issues known to be vital when addressing CVD health (55). Tengland (56) argues that an individualistic lens of behavioural change can limit understandings of a person's CVD health. The result can be too narrow, as the “secondary” effects of their wider environmental conditions (i.e., powerlessness, lack of control, or lack of hope), are not considered. They further suggest that interventions should focus more on the attainment of instrumental goals, such as increased real opportunities in life. For community-based projects to grow further, they should seek to become multi-faceted by combining individualistic interventions with environment/community activities such as community education (57).

The frequency with which mental health issues were raised in discussions was notable. Those who took part in the screening reported high levels of stress and depression, and rates were even higher amongst participants taking part in the programme, furthermore, participants reported lower levels of stress and depression at the end. This may show that this type of intervention is particularly well suited to people with mental health concerns for whom talking to someone can make a real difference. This was also observed in the SPICES consortium partner sites including Brest (France) (58), and Antwerp (Belgium) (59). Most non-specialist or non-clinical people do not think of their health siloed into CVD, mental health, digestive health etc (60). Instead, one's health is perceived holistically, and mental health is often the most prominent barrier and facilitator to behaviour change.

### 9.2 Implementation strategy

We adopted a type 3 hybrid implementation study which focused primarily on implementation factors rather than evaluation, dropping the randomisation approach and embracing flexible more emergent iterative development and growth perspective, co-design, and contextual/place-based factors. A rigid evaluation linear approach as required for a type 1/2 design, which was initially planned, caused tensions with the community-based, participatory, and “emergent” aspects of the project and (2) the pressures imposed on our voluntary sector partners by the pandemic meant that adhering to a rigid randomisation approach was less realistic (7). The planned approach placed power in the hands of the research team which negatively affected our stakeholder relationships, and a rigid adherence to study protocols would have meant we could not effectively adapt strategies or interventions to context.

Instead we adopted a type 3 approach, which has been used to assess a wide range of preventative health and eHealth interventions which operate in communities based on participatory principles (61). In their systematic review of such strategies to implement interventions, Haldane et al. (62) highlight the importance of building mutually beneficial and trust-based relationships particularly with marginalised stakeholders, and stress the importance of developing strategies and interventions contextually whilst reporting and acting on lessons learnt throughout the project. Wildman et al. (63) argue that successful community-based projects require extensive community input, learning and adaptation captured from existing programmes to facilitate the replicability of programmes in other community contexts. With the more flexible type three approach we were able to make local adaptation to meet the need and priorities of the local community and local VCSE partner organisation thereby listening to the voices of those who are involved. This iterative approach to intervention design is similar to the “scaling-out” approach suggested by Aaron et al. (64) which advocates iterative roll-out and local adaptation in place of simply “copy and pasting” interventions across context. In reality, during SPICES-Sussex the local adaption became less flexible as the intervention became more well-developed as the internal factors became more institutionalised within the research team. However, the principle of meeting the needs and priorities of the local VCSE organisation were maintained from site to site and the team sought input from local organisations where possible.

We do not know whether the changes observed will be maintained due to the short follow up period, both at an individual level or a setting level (65), and the research lacks an economic appraisal. The short follow-up period was forced on the research team because of delays to the project caused by COVID-19 which meant our funding period was not long enough to conduct a follow up assessment. An economic appraisal was not considered appropriate because the development approach taken during the study meant that any economic appraisal was not likely to reflect real-world roll-out. In the future we would advocate for greater scaling-out to include a larger sample and an economic appraisal.

### 9.3 Recruitment and retention

The impacts of the restrictions placed on the people, organisations, and communities involved in this research due to COVID-19 were extensive and wide-ranging. The per-implementation phase of the research began in January of 2020 with the recruitment of an implementation team and participant recruitment was due to begin in April 2020. Following the outbreak of COVID-19 in the UK, recruitment was stopped from 16 March 2020 to 1 October 2020. By June 2020, a decision was made to fully move to remote delivery of the coaching intervention using video conferencing services.

Research recruitment and retention were near constant challenges, and all activities were significantly impacted by the Covid restrictions. We believe that the use of the INTERHEART tool, presented on the REDCap platform, acted as a barrier to recruitment as evidenced through the follow sources: (1) Over 650 participants attempted to begin the screening questionnaire and our records show those who did not complete it stopped towards the beginning or mid-way through the questions, particularly when they were asked to measure their waist/hip circumference, (2) of the 380 participants who completed the survey only approximately 100 were eligible for the intervention meaning we were selecting from a very limited pool of participants, (3) many of the participants in the per implementation interviews mentioned finding the screening tool to be “clunky” or “annoying” to use. Its overly “medical” focus, as a basis for lifestyle discussions may not have been engaging for the target audience.

Our initial recruitment strategy was to rely heavily on our VCSE partners to act as gatekeepers for recruitment, a practice commonly seen in participatory research methods (66). Whilst the VCSE partners were adept in the recruitment and management of CHWs and in the development of practices and policies, they did not seem to have the reach or access for the recruitment of large numbers of potential participants. Our experience aligns with that of Williams (67), who states that VCSE and end users' relationships are often smaller in number but deep, based on trust and protection, and covered by a range of risk related policies. Instead, we relied heavily on the use of paid for social media adverts for recruitment due to our ethics restrictions. Much like the experience of other researchers who used these tools, we found that they were low cost and reached large numbers of people but engagement with the screening and risk profiling and participant recruitment was low (68). In future studies, it may be more suitable to use social media as an adjunct to mixed recruitment strategies which make use of community outreach, primary care recruitment, and media outreach (69, 70).

The study sample was heavily skewed towards middle-aged females and much of the sample was not considered to be from vulnerable or low socio-economic groups. Furthermore, males are under-represented in both the risk profiling and intervention samples which represents a divergence with our planned recruitment targets in which we aimed for a more representative sample. The difficulties in recruiting men and vulnerable and other “seldom heard” populations to life style interventions are well-recognised (71, 72). Recommended strategies to improve male participation in community-based interventions include engaging with male-friendly spaces, workplace-based interventions, and incorporating activity-based programming, social-support, and group activities (73, 74). Some of these elements were suggested during the planning phase of SPICES but were not feasible due to COVID restrictions (30).

### 9.4 Project infrastructure

We made the key decision to bring VCSE organisations into the research team with paid roles to foster stronger community/research partnerships as promoted by CBPR researchers (75) and the NHS's PPIE (Public and Patient Involvement and Engagement) initiatives (76). Our research shows that the VCSE sector is an untapped resource within primary and community care that has a great deal of expertise, compassion, and enthusiasm to offer health provision (77). To facilitate this community-based project, we focused on the concept of trust building throughout the intervention as described by Christopher et al. (78).

VCSE partnerships brought knowledge and expertise of their local communities, policies/practices of volunteer management and, critically, perspectives of the motivations and drivers for CHWs and communities. CHWs were empowered to bring their own skills and abilities to the intervention through an asset based and flexible project development which included them in the co-design of the project (79). The strategies we used to implement the interventions were not prescriptive and did not force CHWs to follow a set of strict guidelines. This led to a highly personalised, flexible, and reflective experience for CHWs. However, our experience highlights potential problems with relying on unpaid volunteers to deliver complex interventions, including issues with volunteer commitment, attendance and drop out.

Our research highlights the importance of infrastructure when managing CHWs and partnering with VCSE sector organisations. We developed a bespoke behaviour change training course for CHWs, a range of CHW risk appraisal and mitigation policies with our VCSE partners, and a dedicated team of participant and CHW support and management coordinators. Clear protocols were developed and followed for the recruitment, onboarding, matching, and hosting of participant coaching sessions whilst CHWs were provided with multiple channels of regular communication and continuous training and feedback opportunities. We support calls for project managers, VCSEs, primary care providers, and community members to be more explicitly involved in the design and development of interventions which affect and include communities (80).

In this study, the research team also experienced issues of positionality throughout the project whereby the lines between implementor, community worker, and evaluator were blurred. Coulter et al. (81) have pointed out that research that includes CHWs in the design and delivery of interventions commonly experience a tension between fidelity of the intervention protocol and community expectations, needs, and norms. We also experienced differing goals between academic and community partners (including CHWs), where academic partners prioritized data collection and community partners prioritized funding, sustainability, and policy. This can be likened to the experience of Furman et al. (82) who discussed how community partners were hesitant to endorse their research due to conflicts with on-the-ground realities of the community members they served.

### 9.5 Recommendations

During this project the research team, VCSE partners, and CHWs constantly learnt lessons and were quick to make adaptations to their approach based on feedback from a range of stakeholders and capturing all of these in this paper would be an impossible task. However, several key insights can be drawn from our collective experience and evaluation of the project. They include:

1. Environmental issues are larger and more complex than any coaching intervention based on individualistic changes can hope to remedy.
2. The voluntary and community sector has a range of strengths and assets based on local experience and knowledge developed over significant periods of time that can be used for CVD prevention. However, the sector is highly under-resourced and spread thinly across a wide range of priorities. Individual VCSE partner organisations do not always have enough reach to facilitate recruitment.
3. Community engagement works best if it is built into a project early on through co-design and resources and time should be allocated to this activity.
4. CHWs bring significant advantages during the delivery of community-based interventions. They are trusted peers, they bring their own skills and experience, and they can benefit from the intervention alongside the participants.
5. Strategies to encourage the participation of men should be specifically considered during the planning phase.
6. Virtual coaching interventions are acceptable to participants, and in many cases preferable to participants, due to their flexibility and ease of use.
7. The issue of mental health must be addressed even when working with unrelated health public conditions.
8. A strong project infrastructure, made up of well-trained support/administrative staff, is essential when delivering community-based interventions.
9. CHWs should be paid or reimbursed for their involvement in research and public health interventions. Falling to do so is looked down on my stakeholders and has impacts on sustainability and effectiveness.
10. The Global North can look to innovations in the Global South for examples of success for community-based interventions, however, proper contextual or situational analyses must be conducted to understand the needs and priorities of target communities.

### 9.6 Conclusion

This study demonstrates the feasibility of a CHW-led preventative health interventions could be implemented with overseen and unheard communities in the UK. It highlights the wealth of untapped resources that exist with VCSE and CHWs and suggests how a beneficial community-based service could be set up to run alongside and support NHS Health Checks, to reduce the incidence of CVD. The aim was to empower CHWs to discuss health with people in their communities based on behaviour change principles. We have set out what worked well and what did not, to facilitate development of future community-based interventions in the Global North. We believe that the community-based approach need not be restricted to CVD risk reduction, and that it could easily be applied to low level mental health conditions, diabetes, or other preventable NCDs. If CHWs are confident, well supported, and well-trained, they will have the skills and ability to contribute to improving the health and wellbeing of people in their communities. The benefits do not only extend to patients but also to CHWs and to the VCSE partners involved. We believe our project shows how these interventions can become a supplementary tool that links primary care services with the VCSE sector.

## Data Availability

The datasets presented in this study can be found in the University of Sussex's data repository through the following link: https://sussex.figshare.com/; (doi: 10.25377/sussex.25569084).

## References

[1] World Health Organisation. Cardiovascular Diseases Fact Sheet. (2022) (cited Nov 7, 2022). Cardiovascular diseases Fact Sheet. Available online at: https://www.who.int/health-topics/cardiovascular-diseases#tab=tab_1

[2] Matsushita K Ding N Kou M Hu X Chen M Gao Y The relationship of COVID-19 severity with cardiovascular disease and its traditional risk factors: a systematic review and meta-analysis. Glob Heart. (2020) 15(1). 10.5334/gh.814 33150129 PMC7546112

[3] National Institute for Health and Care Excellence. Prevention of Cardiovascular disease (PH25)—Review Proposal (2014).

[4] Marsh K Phillips CJ Fordham R Bertranou E Hale J. Estimating cost-effectiveness in public health: a summary of modelling and valuation methods. Health Econ Rev. (2012) 2(1):1–6. 10.1186/2191-1991-2-17 22943762 PMC3484026

[5] Brush BL Mentz G Jensen M Jacobs B Saylor KM Rowe Z Success in long-standing community-based participatory research (CBPR) partnerships: a scoping literature review. Health Educ Behav. (2020) 47(4):556–68. 10.1177/1090198119882989 31619072 PMC7160011

[6] Bogart LM Uyeda K. Community-based participatory research: partnering with communities for effective and sustainable behavioral health interventions. Health Psychol. (2009) 28(4):391–3. 10.1037/a0016387 19594261 PMC2854509

[7] Thomas P. Collaborating for Health. London: Routledge (2017).

[8] Hassen HY Bowyer M Gibson L Abrams S Bastiaens H. Level of cardiovascular disease knowledge, risk perception and intention towards healthy lifestyle and socioeconomic disparities among adults in vulnerable communities of Belgium and England. BMC Public Health. (2022) 22(1):1–9. 10.1186/s12889-021-12274-7 35093056 PMC8800212

[9] Hassen HY Ndejjo R Musinguzi G Van Geertruyden JP Abrams S Bastiaens H. Effectiveness of community-based cardiovascular disease prevention interventions to improve physical activity: a systematic review and meta-regression. Prev Med. (2021) 153:106797. 10.1016/j.ypmed.2021.106797 34508731

[10] Robson J Dostal I Sheikh A Eldridge S Madurasinghe V Griffiths C The NHS health check in England: an evaluation of the first 4 years. BMJ Open. (2016) 6(1):e008840. 10.1136/bmjopen-2015-008840 26762161 PMC4735215

[11] Office for Improvement and Disapities, UK Government. NHS Health Checks: Applying All Our Health. (2022). Available online at: https://www.gov.uk/government/publications/nhs-health-checks-applying-all-our-health/nhs-health-checks-applying-all-our-health (Accessed January 25, 2023).

[12] Martin A Saunders CL Harte E Griffin SJ MacLure C Mant J Delivery and impact of the NHS health check in the first 8 years: a systematic review. Br J Gen Pract. (2018) 68(672):e449–59. 10.3399/bjgp18X697649 29914882 PMC6014431

[13] Tanner L Kenny RPW Still M Ling J Pearson F Thompson K NHS health check programme: a rapid review update. BMJ Open. (2022) 12(2):e052832. 10.1136/bmjopen-2021-052832 35172998 PMC8852663

[14] Agarwal S Jakes S Essex S Page SJ Mowforth M. Disadvantage in English seaside resorts: a typology of deprived neighbourhoods. Tour Manag. (2018) 69:440–59. 10.1016/j.tourman.2018.06.012

[15] UK Chief Medical Officer. Health in Coastal Communities. UK Chief Medical Officer; (2021). p. 259.

[16] Cyril S Smith BJ Possamai-Inesedy A Renzaho AM. Exploring the role of community engagement in improving the health of disadvantaged populations: a systematic review. Glob Health Action. (2015) 8(1):29842. 10.3402/gha.v8.29842 26689460 PMC4685976

[17] Ndejjo R Hassen HY Wanyenze RK Musoke D Nuwaha F Abrams S Community-based interventions for cardiovascular disease prevention in low-and middle-income countries: a systematic review. Public Health Rev. (2021) 42. 10.3389/phrs.2021.1604018 34692177 PMC8386815

[18] Khetan AK Purushothaman R Chami T Hejjaji V Mohan SKM Josephson RA The effectiveness of community health workers for CVD prevention in LMIC. Glob Heart. (2017) 12(3):233–243. 6. 10.1016/j.gheart.2016.07.001 27993594

[19] Joshi R Peiris D. Task-sharing for the prevention and control of non-communicable diseases. Lancet Glob Health. (2019) 7(6):e686–7. 10.1016/S2214-109X(19)30161-5 31097265

[20] Adams C. Toward an institutional perspective on social capital health interventions: lay community health workers as social capital builders. Sociol Health Illn. (2020) 42(1):95–110. 10.1111/1467-9566.12992 31674684

[21] Van Velsen L Illario M Jansen-Kosterink S Crola C Di Somma C Colao A A community-based, technology-supported health service for detecting and preventing frailty among older adults: a participatory design development process. J Aging Res. (2015) 2015:9–18. 10.1155/2015/216084 PMC4541013 26346580

[22] Michie S Abraham C Whittington C McAteer J Gupta S. Effective techniques in healthy eating and physical activity interventions: a meta-regression. Health Psychol. (2009) 28(6):690. 10.1037/a0016136 19916637

[23] Yardley L Morrison L Bradbury K Muller I. The person-based approach to intervention development: application to digital health-related behavior change interventions. J Med Internet Res. (2015) 17(1):e4055. 10.2196/jmir.4055 PMC4327440 25639757

[24] Person-Based Approach. The Person-Based Approach For Developing Health Interventions. (2022). Available online at: https://www.personbasedapproach.org/#:∼:text=What%20is%20the%20Person%2DBased,and%20experiences%20of%20intervention%20users (Accessed January 25, 2023).

[25] Hopkins T Rippon S. Head, Hands and Heart: Asset-Based Approaches in Health Care. London: Health Foundations (2015).

[26] Barrera M Castro FG Strycker LA Toobert DJ. Cultural adaptations of behavioral health interventions: a progress report. J Consult Clin Psychol. (2013) 81(2):196–205. 10.1037/a0027085 22289132 PMC3965302

[27] Moore GF Audrey S Barker M Bond L Bonell C Hardeman W. process evaluation of complex interventions: medical research council guidance. Br Med J. (2015) 350. 10.1136/bmj.h1258 PMC4366184 25791983

[28] Brown A Ramsay N Milo M Moore M Hossain R. How research-based theatre is a solution for community engagement and advocacy at regional medical campuses: the health and equity through advocacy, research, and theatre (HEART) program. Can Med Educ J. (2018) 9(1):e6. 10.36834/cmej.42191 30140330 PMC6104337

[29] Nahar P Van Marwijk H Gibson L Musinguzi G Anthierens S Ford E A protocol paper: community engagement interventions for cardiovascular disease prevention in socially disadvantaged populations in the UK: an implementation research study. Glob Health Res Policy. (2020) 5:1–9. 10.1186/s41256-020-0131-1 32190745 PMC7068925

[30] Grice-Jackson T Rogers I Ford E Van Marwijk H Topham C Musinguzi G The pre-implementation phase of a project seeking to deliver a community-based CVD prevention intervention (SPICES-Sussex): a qualitative study exploring views and experience relating to intervention development. Health Promot Pract. (2023):15248399231182139. 10.1177/15248399231182139 37386868 PMC11528968

[31] Glasgow RE McKay HG Piette JD Reynolds KD. The RE-AIM framework for evaluating interventions: what can it tell us about approaches to chronic illness management? Patient Educ Couns. (2001) 44(2):119–27. 10.1016/S0738-3991(00)00186-5 11479052

[32] Skivington K Matthews L Simpson SA Craig P Baird J Blazeby JM. A new framework for developing and evaluating complex interventions: update of medical research council guidance. Br Med J. (2021) 374:1–11. 10.1136/bmj.n2061 PMC8482308 34593508

[33] Ministry of Housing, Communities & Local Government UK Government. English Indices of Deprivation 2019. (2019) (cited Nov 7, 2022). Available online at: https://www.gov.uk/government/statistics/english-indices-of-deprivation-2019

[34] Palmer VJ. The participatory zeitgeist in health care: it is time for a science of participation. J Particip Med. (2020) 12(1):e15101. 10.2196/15101 33064092 PMC7434075

[35] McGorrian C Yusuf S Islam S Jung H Rangarajan S Avezum A Estimating modifiable coronary heart disease risk in multiple regions of the world: the INTERHEART modifiable risk score. Eur Heart J. (2011) 32(5):581–9. 10.1093/eurheartj/ehq448 21177699

[36] Harris PA Taylor R Minor BL Elliott V Fernandez M O’Neal L The REDCap consortium: building an international community of software platform partners. J Biomed Inform. (2019) 95:103208. 10.1016/j.jbi.2019.103208 31078660 PMC7254481

[37] Piepoli MF Hoes AW Agewall S Albus C Brotons C Catapano AL Guidelines: editor’s choice: 2016 European guidelines on cardiovascular disease prevention in clinical practice: the sixth joint task force of the European society of cardiology and other societies on cardiovascular disease prevention in clinical practice (constituted by representatives of 10 societies and by invited experts) developed with the special contribution of the European association for cardiovascular prevention & rehabilitation (EACPR). Eur Heart J. (2016) 37(29):2315. 10.1093/eurheartj/ehw106 27222591 PMC4986030

[38] Levounis P Arnaout B Marienfeld C. Motivational interviewing for clinical practice. Arlington: American Psychiatric Association Publishing (2017). p. 15–35.

[39] Miller WR Rollnick S. Motivational Interviewing: Helping People Change. New York: Guilford press (2012).

[40] Harden SM Smith ML Ory MG Smith-Ray RL Estabrooks PA Glasgow RE. RE-AIM in clinical, community, and corporate settings: perspectives, strategies, and recommendations to enhance public health impact. Front Public Health. (2018) 6:71. 10.3389/fpubh.2018.00071 29623270 PMC5874302

[41] Kwan BM McGinnes HL Ory MG Estabrooks PA Waxmonsky JA Glasgow RE. RE-AIM in the real world: use of the RE-AIM framework for program planning and evaluation in clinical and community settings. Front Public Health. (2019) 7:345. 10.3389/fpubh.2019.00345 31824911 PMC6883916

[42] Glasgow RE Askew S Purcell P Levine E Warner ET Stange KC Use of RE-AIM to address health inequities: application in a low-income community health center-based weight loss and hypertension self-management program. Transl Behav Med. (2013) 3(2):200–10. 10.1007/s13142-013-0201-8 23750180 PMC3671594

[43] Onono M Abdi M Opondo I Okung’u J Asadhi E Nyamai R Using the RE-AIM framework to evaluate the implementation of integrated community case management in Kenya. Acta Paediatr. (2018) 107:53–62. 10.1111/apa.14662 30570791

[44] Wippold GM Garcia KA Frary SG Griffith DM. Community health worker interventions for men: a scoping review using the RE-AIM framework. Health Educ Behav. (2023) 51(1):10901981231179498. 10.1177/1090198123117949 37350223

[45] Hagströmer M Oja P Sjöström M. The international physical activity questionnaire (IPAQ): a study of concurrent and construct validity. Public Health Nutr. (2006) 9(6):755–62. 10.1079/PHN2005898 16925881

[46] England CY Thompson JL Jago R Cooper AR Andrews RC. Development of a brief, reliable and valid diet assessment tool for impaired glucose tolerance and diabetes: the UK diabetes and diet questionnaire. Public Health Nutr. (2017) 20(2):191–9. 10.1017/S1368980016002275 27609314 PMC5244439

[47] Musinguzi LK Turinawe EB Rwemisisi JT de Vries DH Mafigiri DK Muhangi D Linking communities to formal health care providers through village health teams in rural Uganda: lessons from linking social capital. Hum Resour Health. (2017) 15:1–16. 10.1186/s12960-016-0177-9 28077148 PMC5225547

[48] Katz E Chikwenhere Y Essien E Olirus Owilli A Westerhaus M. Rethinking global health from south and north: a social medicine approach to global health education. Glob Public Health. (2023) 18(1):2191685. 10.1080/17441692.2023.2191685 36947564

[49] Anand TN Joseph LM Geetha AV Chowdhury J Prabhakaran D Jeemon P. Task-sharing interventions for cardiovascular risk reduction and lipid outcomes in low-and middle-income countries: a systematic review and meta-analysis. J Clin Lipidol. (2018) 12(3):626–42. 10.1016/j.jacl.2018.02.008 29559305 PMC5994347

[50] Ndejjo R Wanyenze RK Nuwaha F Bastiaens H Musinguzi G. Barriers and facilitators of implementation of a community cardiovascular disease prevention programme in Mukono and Buikwe districts in Uganda using the consolidated framework for implementation research. Implement Sci. (2020) 15:1–17. 10.1186/s13012-020-01065-0 33298098 PMC7726905

[51] Haines A de Barros EF Berlin A Heymann DL Harris MJ. National UK programme of community health workers for COVID-19 response. Lancet. (2020) 395(10231):1173–5. 10.1016/S0140-6736(20)30735-2 32220277 PMC7146683

[52] Ballard M Odera M Bhatt S Geoffrey B Westgate C Johnson A. Payment of community health workers. Lancet Glob Health. (2022) 10(9):e1242. 10.1016/S2214-109X(22)00311-4 35961344

[53] Hanson K Brikci N Erlangga D Alebachew A De Allegri M Balabanova D The lancet global health commission on financing primary health care: putting people at the centre. Lancet Glob Health. (2022) 10(5):e715–72. 10.1016/S2214-109X(22)00005-5 35390342 PMC9005653

[54] NIHR. UK Standards for Public Involvement. (2024) (cited Jan 9, 2024). Available online at: https://sites.google.com/nihr.ac.uk/pi-standards/home

[55] Schmid TL Pratt M Howze E. Policy as intervention: environmental and policy approaches to the prevention of cardiovascular disease. Am J Public Health. (1995) 85(9):1207–11. 10.2105/AJPH.85.9.1207 7661226 PMC1615578

[56] Tengland PA. Behavior change or empowerment: on the ethics of health-promotion goals. Health Care Anal. (2016) 24(1):24–46. 10.1007/s10728-013-0265-0 24100936

[57] Pennant M Davenport C Bayliss S Greenheld W Marshall T Hyde C. Community programs for the prevention of cardiovascular disease: a systematic review. Am J Epidemiol. (2010) 172(5):501–16. 10.1093/aje/kwq171 20667932

[58] Le Goff D Barais M Perraud G Derriennic J Aujoulat P Guillou-Landreat M Innovative cardiovascular primary prevention population-based strategies: a 2-year hybrid type 1 implementation randomised control trial (RCT) which evaluates behavioural change conducted by community champions compared with brief advice only from the SPICES project (scaling-up packages of interventions for cardiovascular disease prevention in selected sites in Europe and sub-Saharan Africa). BMC Public Health. (2021) 21(1):1–9. 10.1186/s12889-021-11443-y 34281516 PMC8287807

[59] Aerts N Anthierens S Van Bogaert P Peremans L Bastiaens H. Prevention of cardiovascular diseases in community settings and primary health care: a Pre-implementation contextual analysis using the consolidated framework for implementation research. Int J Environ Res Public Health. (2022) 19(14):8467. 10.3390/ijerph19148467 35886317 PMC9323996

[60] Collyer TA Smith KE. An atlas of health inequalities and health disparities research:“how is this all getting done in silos, and why?”. Soc Sci Med. (2020) 264:113330. 10.1016/j.socscimed.2020.113330 32971486 PMC7449896

[61] Van Velsen L Wentzel J Van Gemert-Pijnen JE. Designing eHealth that matters via a multidisciplinary requirements development approach. JMIR Res Protoc. (2013) 2(1):e2547. 10.2196/resprot.2547 PMC3815432 23796508

[62] Haldane V Chuah FL Srivastava A Singh SR Koh GC Seng CK Community participation in health services development, implementation, and evaluation: a systematic review of empowerment, health, community, and process outcomes. PLoS One. (2019) 14(5):e0216112. 10.1371/journal.pone.0216112 31075120 PMC6510456

[63] Wildman JM Valtorta N Moffatt S Hanratty B. ‘What works here doesn’t work there’: the significance of local context for a sustainable and replicable asset-based community intervention aimed at promoting social interaction in later life. Health Soc Care Community. (2019) 27(4):1102–10. 10.1111/hsc.12735 30864266 PMC6849711

[64] Aarons GA Sklar M Mustanski B Benbow N Brown CH. “Scaling-out” evidence-based interventions to new populations or new health care delivery systems. Implement Sci. (2017) 12:1–13. 10.1186/s13012-016-0533-0 28877746 PMC5588712

[65] Luten KA Reijneveld SA Dijkstra A de Winter AF. Reach and effectiveness of an integrated community-based intervention on physical activity and healthy eating of older adults in a socioeconomically disadvantaged community. Health Educ Res. (2016) 31(1):98–106. 10.1093/her/cyv064 26675175 PMC4883033

[66] Goodman A Gatward R. Who are we missing? Area deprivation and survey participation. Eur J Epidemiol. (2008) 23(6):379–87. 10.1007/s10654-008-9248-0 18409005

[67] Williams P. “It all sounds very interesting, but we’re just too busy!”: exploring why “gatekeepers” decline access to potential research participants with learning disabilities. Eur J Spec Needs Educ. (2020) 35(1):1–14. 10.1080/08856257.2019.1687563

[68] McRobert CJ Hill JC Smale T Hay EM Van der Windt DA. A multi-modal recruitment strategy using social media and internet-mediated methods to recruit a multidisciplinary, international sample of clinicians to an online research study. PLoS One. (2018) 13(7):e0200184. 10.1371/journal.pone.0200184 29979769 PMC6034855

[69] Khatri C Chapman SJ Glasbey J Kelly M Nepogodiev D Bhangu A Social media and internet driven study recruitment: evaluating a new model for promoting collaborator engagement and participation. PloS one. (2015) 10(3):e0118899. 10.1371/journal.pone.0118899 25775005 PMC4361707

[70] UyBico SJ Pavel S Gross CP. Recruiting vulnerable populations into research: a systematic review of recruitment interventions. J Gen Intern Med. (2007) 22(6):852–63. 10.1007/s11606-007-0126-3 17375358 PMC2219860

[71] Shaghaghi A Bhopal RS Sheikh A. Approaches to recruiting “hard-to-reach”populations into research: a review of the literature. Health Promot Perspect. (2011) 1(2):86. 10.5681/hpp.2011.009 24688904 PMC3963617

[72] Pagoto SL Schneider KL Bodenlos JS Appelhans BM Whited MC Ma Y Association of post-traumatic stress disorder and obesity in a nationally representative sample. Obesity. (2012) 20(1):200–5. 10.1038/oby.2011.318 22016096

[73] Oliffe JL Rossnagel E Bottorff JL Chambers SK Caperchione C Rice SM. Community-based men’s health promotion programs: eight lessons learnt and their caveats. Health Promot Int. (2020) 35(5):1230–40. 10.1093/heapro/daz101 31603471

[74] Bell OJ Flynn D Clifford T West D Stevenson E Avery L. Identifying behavioural barriers and facilitators to engaging men in a community-based lifestyle intervention to improve physical and mental health and well-being. Int J Behav Nutr Phys Act. (2023) 20(1):1–14. 10.1186/s12966-022-01404-y 36879249 PMC9990339

[75] Springer MV Skolarus LE. Community-based participatory research: partnering with communities. Stroke. (2019) 50(3):e48–50. 10.1161/STROKEAHA.118.024241 30661505 PMC6594378

[76] Holmes L Cresswell K Williams S Parsons S Keane A Wilson C Innovating public engagement and patient involvement through strategic collaboration and practice. Res Involvement Engagem. (2019) 5(1):1–12. 10.1186/s40900-019-0160-4 PMC6802177 31646001

[77] Ragsdell G. Voluntary sector organisations: untapped sources of lessons for knowledge management. Proceedings of the International Conference on Intellectual Capital, Knowledge Management & Organizational Learning (2013). p. 349–55

[78] Christopher S Watts V McCormick AKHG Young S. Building and maintaining trust in a community-based participatory research partnership. Am J Public Health. (2008) 98(8):1398–406. 10.2105/AJPH.2007.125757 18556605 PMC2446462

[79] Bainbridge L Lunt N. Place, strengths and assets: a case study of how local area coordination is supporting individuals and families under conditions of austerity. Br J Social Work. (2021) 51(4):1354–73. 10.1093/bjsw/bcab041

[80] Di Maddaloni F Davis K. Project manager’s perception of the local communities’ stakeholder in megaprojects. An empirical investigation in the UK. Int J Proj Manag. (2018) 36(3):542–65. 10.1016/j.ijproman.2017.11.003

[81] Coulter K Ingram M McClelland DJ Lohr A. Positionality of community health workers on health intervention research teams: a scoping review. Front Public Health. (2020) 8:208. 10.3389/fpubh.2020.00208 32612967 PMC7308474

[82] Furman L Matthews L Davis V Killpack S O’Riordan MA. Breast for success: a community–academic collaboration to increase breastfeeding among high-risk mothers in Cleveland. Prog Community Health Partnersh. (2016) 10(3):341–53. 10.1353/cpr.2016.0041 28230542

