# Supplementary material for: A community health worker led approach to cardiovascular disease prevention in the UK—SPICES-Sussex (scaling-up packages of interventions for cardiovascular disease prevention in selected sites in Europe and Sub-saharan Africa): an implementation research project
Source: Front Health Serv. 2024 May 7;4:1152410. doi: 10.3389/frhs.2024.1152410 (PMC11113076; doi:10.3389/frhs.2024.1152410)
Supplement: Supplementary file 1 [file Table1.docx]

| **Appendix 1:** Descriptive statistics for risk profiling for participants who were screened for the intervention, those that withdrew from the intervention and those that completed the intervention using the Interheart survey. | | | | | |
| --- | --- | --- | --- | --- | --- |
|  | **Completed screening questionnaire (n=381)** | **Started but withdrew n=17** | **Completed intervention n=29 (data shown for participants with complete data n=27)** | | **p value** |
|  |  |  | **Pre-Intervention** | **Post intervention** |  |
| Sex |  |  |  |  |  |
| *Male* n (%) | 71 (22.9%) | 1 (6%) | 4 (14%) | 4 (14%) | - |
| Female n (%) | 310 | 16 | 23 (86%) | 23 (86%) | - |
| Risk Category |  |  |  |  |  |
| Low | 179 (47.6%) | -l | - *NB* | - | - |
| Moderate | 104 (27.7%) | - | - *NB* | - | - |
| High | 93 (24.7%) | -k | - *NB* | - | - |
| Research Site |  |  |  |  |  |
| Newhaven (%) | -- *NB* | - | 9 | 10 | - |
| East Brighton (%) | -- *NB* | - | 7 | 6 | - |
| Hastings (%) | -- *NB* | - | 6 | 5 | - |
| Hangleton (%) | -- *NB* | - | 7 | 6 | - |
| Age (yrs, SD) | 58 (12.39) | - | 58 (11.94) | 58 (11.94) | - |
| INTERHEART (m, SD) | 8.8 (18.71) | - | 11.7 (1.70) | 9.89 (3.23)^1^ | .009* |
| Family History^a^ | 113 (29%) | - | 9 (33%) | 9 (33%) | - |
| Diabetes^a^ | 21 (6%) | - | 1 (4%) | 1 (4%) | - |
| Blood pressure^a^ | 88 (22%) | - | 5 (19%) | 4 (15%) | - |
| Smoke^b^ | 216 (56%) | - | 1 (3.7%) | 1 (4%) | - |
| Stress^c^ | 233 (61%) | - | 21 (78%) | 17 (63%) | - |
| Depression^c^ | 161 (41%) | - | 13 (48%) | 10 (37%) | - |
| Salty foods^d^ | 169 (44%) | - | 10 (37%) | 6 (22%) | - |
| Deep fried food^d^ | 58 (15%) | - | 4 (14%) | 3 (11%) | - |
| Fruit p/ day ^e^ | 87 (23%) | - | 5 (19%) | 3 (11%) | - |
| Veg p/ day^e^ | 45 (11%) | - | 3 (11%) | 2 (7%) | - |
| Meat p/day^d^ | 93 (24%) |  | 7 (25%) | 8 (29.6%) | - |
| Physical inactivity^f^ | 212 (55%) | - | 17 (64%) | 10 (37%) | - |
| RoC (m, SD)^g^ | 3.5 (.95) | - | 3.6 (.92) | 4.5 (.64)^1^ | - |
| UKDDQ |  |  |  |  |  |
| Wholegrains^h^ | - | - | 62% (29) | 75% (25)^1^ | .02* |
| Fruit and Veg^i^ | - | - | 3.42 (2.01) | 4.46 (2.18) ^1^ | .005* |
| Sugary foods^j^ | - | - | 13.85 (15.32) | 8.38 (6.89) ^1^ | .01* |
| Fatty foods^j^ | - | - | 11.31 (7.55) | 9.33 (5.88) ^1^ | .03* |
| Salty foods^j^ | - | - | 6.95 (3.76) | 5.12 (2.43) ^1^ | .004* |
| Oily Fish^k^ | - | - | 56% | 59% | 1^2^ |
| IPAQ |  |  |  |  | .004*^2^ |
| Low | - | - | 11 (40.7%) | 2 (7.4%) | - |
| Medium/High | - | - | 16 (59.3) | 25 (92.6%) | - |

*a=did have diabetes/HBP/family history of CVD, b=is a current smoker, c=does experience stress/depression, d=does consume meat twice daily/ deep fried food/salty foods frequently, e=does not consume fruit/vegetables regularly, f= is mainly sedentary, g= readiness to make lifestyle changes (1-5 scale, 5 =more ready to make changes), h= percentage choosing wholegrain cereals, i = portions per day, j= portions per week, k= percentage eating oily fish at least once per week. NB Participants were not organised into research sites until after the risk profiling stage and* *all participants in the intervention were in the moderate risk category*. *1=Wilcoxon sign test, 2= McNemar’s test,* **p*<0.05*. Note, the 2 participants who completed the intervention but who did not complete the post evaluation survey are not included in table 1. Note: 29 participants completed the intervention, but data is presented for only 27 participants as 2 participants completed the intervention but did not provide full or usable data for the analysis.*

| **Appendix 2:** Data on implementation fidelity and retention rates monitoring the number of sessions completed by participants at each study site. | | | | | | | | |
| --- | --- | --- | --- | --- | --- | --- | --- | --- |
| **Site** | **Eligible** | **Consent (*)** | **1(**)** | **2(***)** | **3(***)** | **4(***)** | **5(***)** | **6(***)** |
| Hastings | 33 | 17 (52%) | 14 (82%) | 9 (64%) | 6 (43%) | 5 (38%) | 5 (38%) | 4 (31%) |
| Hangleton | 22 | 12 (55%) | 11 (92%) | 10 (91%) | 7 (64%) | 6 (55%) | 5 (45%) | 4 (36%) |
| Newhaven | 32 | 12 (38%) | 12 (100%) | 11 (92%) | 9 (75%) | 8 (73%) | 7 (64%) | 7 (63%) |
| East Brighton | 19 | 10 (53%) | 9 (90%) | 9 (100%) | 7 (77%) | 6 (75%) | 6 (75%) | 5 (63%) |
| **Total** | **106** | **51 (48%)** | **46 (90%)** | **39 (84%)** | **29 (63%)** | **25 (54%)** | **23(50%)** | **21 (45%)** |

** = Percentage of eligible participants who consented to take part in the study during an induction meeting, Eligible participants were defined as those who completed the risk profiling questionnaire at a medium risk level. **= Percentage of consenting participants who took part in the first CHW coaching session. ***= Percentage of those who took part in their first CHW coaching session who attended subsequent CHW coaching sessions.*

| **Appendix 3.** Key changes to the study design compared with protocol paper (29). The COVID lockdowns in 2020 resulted in several changes to the study design from the original SPICES Sussex protocol |
| --- |
| 1. *Pause in implementation:* The project was due to begin its implementation phase in April 2020 but was put on pause due the first lockdown. No data collection or recruitment of participants was carried out until October 2020; however, volunteer recruitment and intervention co-design were conducted from May to August 2020. |
| 1. *Online implementation:* Due to COVID restrictions the decision was made in September 2020 to move the project to an entirely online coaching intervention. This meant the removal of the option of in person group coaching sessions, and the adoption of virtual one-on-one coaching sessions delivered over Zoom. Volunteer training was also conducted online. |
| 1. *Removal of stepped-wedge randomization:* Feedback received during the strategy co-design process, that a flexible, emergent approach sits poorly with randomisation, and practical difficulties involved in recruiting and managing VCSEs and CHWs during the COVID lockdowns, meant that in April 2020 the decision was made to remove randomization. |
| 1. *Changes to the schedule of coaching sessions:* The protocol was for participants to receive monthly coaching sessions for six months. However, because of the compressed time for the study (see section 3.1 for more details), some participants at the later-to-join sites (East Brighton and Newhaven) held coaching sessions at shorter intervals, to allow them to complete all six sessions before the end of the study timeframe. |
| 1. *Dropping of post-intervention follow-up questionnaires:* The original plan was for questionnaire measures of target behaviours and CVD risk to be repeated 3-monthly at multiple timepoints after the intervention to assess whether behaviour change had been sustained. Due to COVID-related delays to participant recruitment this follow-up was no longer possible within the study timeframe. |
